# Effectiveness of xenogenous-based bovine-derived platelet gel embedded within a three-dimensional collagen implant on the healing and regeneration of the Achilles tendon defect in rabbits

**DOI:** 10.1517/14712598.2014.915305

**Published:** 2014-05-19

**Authors:** Ali Moshiri, Ahmad Oryan, Abdolhamid Meimandi-Parizi, Omid Koohi-Hosseinabadi

**Affiliations:** ^a^ Division of Surgery and Radiology, Department of Clinical Sciences, School of Veterinary Medicine, Shiraz University, Shiraz, Iran+98 9123409835; dr.ali.moshiri@gmail.com; ^b^ Department of Pathology, School of Veterinary Medicine, Shiraz University, Shiraz, Iran; ^c^ Division of Surgery and Radiology, Department of Clinical Sciences, School of Veterinary Medicine, Shiraz University, Shiraz, Iran; ^d^ Center of Comparative and Experimental Medicine, Shiraz University of Medical Sciences, Shiraz, Iran

**Keywords:** bovine platelet, healing, regenerative medicine, tendon, tissue engineering

## Abstract

***Background and objective:*** Tissue engineering is an option in reconstructing large tendon defects and managing their healing and regeneration. We designed and produced a novel xenogeneic-based bovine platelet, embedded it within a tissue-engineered collagen implant (CI) and applied it in an experimentally induced large tendon defect model in rabbits to test whether bovine platelets could stimulate tendon healing and regeneration *in vivo.*

***Methods:*** One hundred twenty rabbits were randomly divided into two experimental and pilot groups. In all the animals, the left Achilles tendon was surgically excised and the tendon edges were aligned by Kessler suture. Each group was then divided into three groups of control (no implant), treated with CI and treated with collagen-platelet implant. The pilot groups were euthanized at 10, 15, 30 and 40 days post-injury (DPI), and their gross and histologic characteristics were evaluated to study host–graft interaction mechanism. To study the tendon healing and its outcome, the experimental animals were tested during the experiment using hematologic, ultrasonographic and various methods of clinical examinations and then euthanized at 60 DPI and their tendons were evaluated by gross pathologic, histopathologic, scanning electron microscopic, biophysical and biochemical methods.

***Results:*** Bovine platelets embedded within a CI increased inflammation at short term while it increased the rate of implant absorption and matrix replacement compared with the controls and CI alone. Treatment also significantly increased diameter, density, amount, alignment and differentiation of the collagen fibrils and fibers and approximated the water uptake and delivery behavior of the healing tendons to normal contralaterals (p *<* 0.05). Treatment also improved echogenicity and homogenicity of the tendons and reduced peritendinous adhesion, muscle fibrosis and atrophy, and therefore, it improved the clinical scores and physical activity related to the injured limb when compared with the controls (p *<* 0.05).

***Conclusion:*** The bovine platelet gel embedded within the tissue-engineered CI was effective in healing, modeling and remodeling of the Achilles tendon in rabbit. This strategy may be a valuable option in the clinical setting.

## 1. Introduction

Large Achilles tendon defect is a considerable challenge and usually happens due to traumas, tendinopathies, tumors, ischemia and necrosis, gangrenous and infective ulcers, fourth-degree burnings, neglected tendon ruptures and several other reasons that require excision of a considerable amount of the injured tendon [1-3]. Tendon defects should be reconstructed to restore the function at short term and to assist new tendon formation, healing and regeneration, at mid to long term [4]. Although many surgical techniques have been tried to reconstruct large tendon defects, all these methods have limitations [2,4,5]. In addition, auto-, allo- and xenografts as tendon substitutes have limitations [6,7]. Donor site morbidity, pain, insufficient amount, increase in surgical time and requiring double surgery are limitations of autografts. In contrast, using allo- and xenografts has the risks of disease transmission, lower healing capability and higher risk of rejection [3,5].

Tissue engineering and regenerative medicine is a newer option than classic methods [8,9]. The goal is to produce a viable graft to be considered as a replacement of autografts [10]. Scaffolds are the first step used to reconstruct tissue defects and to promote tissue healing and regeneration [4,11]. Collagen is one of the best biomaterial for tendon tissue engineering and producing the scaffolds [12-14]. As the second step, equipment of the scaffolds with healing promotive factors is essential to modulate and promote several events during tendon healing [4,15,16].

Growth factors have important roles in tendon healing [4,17-19]. They regulate cellular behaviors such as migration, proliferation, differentiation, maturation and matrix production. They also regulate differentiation of the collagen fibril to fiber and fiber bundle and result in tissue alignment, maturation and remodeling [4,20]. All these events are necessary in optimizing the tendon healing [4]. Although application of growth factors is beneficial in tendon healing, they are very expensive, which limits their clinical application [19]. A cost-effective and a newer source of growth factors have been introduced.

Platelets have several growth factors and pro-inflammatory mediators [20-23]. These growth factors are reserved in α-granules. The α-granules are also sources of cytokines, chemokines and many other proteins variously involved in stimulating chemotaxis, cell proliferation and maturation, modulating the inflammatory molecules and attracting the leukocytes [19]. In addition, platelets store antibacterial and fungicidal proteins to prevent infections and proteases such as metalloprotease-4 and coagulation factors [4,21]. Platelets also contain dense granules, which store and release, upon activation, ADP, ATP, calcium ions, histamine, 5-hydroxytryptamine and dopamine [4,24]. Moreover, platelets contain lysosomal granules that can secrete acid hydrolases, cathepsin D and E, elastase and lysozyme and most likely other not yet well-characterized molecules, whose role in the process of tissue healing should not be underestimated [25,26].

They could be extracted from peripheral blood of the patients (autograft source) or could be harvested from other sources (allograft or xenograft sources) [27-29]. The most famous form of platelets, have been widely used in the clinics, is platelet-rich plasma (PRP), which could be produced by a one- or two-step centrifugation. The differences between the PRPs are related to the concentration of the platelets and the presence or absence of white blood cells (WBCs) and red blood cells (RBCs) [30]. Leukocytes play a key role not only in the initial phases of inflammation but have also been reported to exaggerate the inflammation and the potential for localized pain [25]. As RBCs have limited role in tendon healing, and the presence of them in PRP increases the tendency for spontaneous platelet clumping and/or aggregation, it is advised to remove them from the PRP solution. In addition, erythrocyte lysis releases free radicals that harm tissue structures [31]. The effectiveness of PRP is dependent on their preparation method and platelet concentration [32].

A concentration of three to seven times more than the blood platelet concentration has been suggested to be effective *in vivo* [19]. However, more concentrations have been claimed to have negative effect on tendon healing [30]. Platelets are not activated in the PRP, but after injection in the injured area and exposure to the injured collagen fibers and cells, they become activated, thus releasing their growth factors and mediators [19]. Platelet gel (PG) could be produced by activation of the PRP out of the patient body using bovine thrombin and CaCl or other methods [22]. PG has better availability after filling the defect area and may be more effective in healing and regeneration, than PRP [33]. However, there is no general agreement on this issue.

Recent studies suggest that other sources of platelets (e.g., allo- and xenografts) may be at least as effective as autografts [27,34,35]. It has been suggested that platelets have superior efficacy in animal-based studies, whereas lower efficacy has been documented in the human clinical studies [19,36]. This important difference may correlate with the source of the platelets. In the animal studies, allograft source has been used mostly, whereas in the clinical studies, autograft source has been used [27,28,34,37,38]. This is the area of debate [19]. The higher efficacy of the allograft platelets over the autograft type may correlate with the superiority of the allograft source in increasing the inflammation [19]. The role of well-produced inflammatory response but not the exaggerated one in healing and regeneration has been well demonstrated, previously [6,7]. In addition, recent investigations suggest that xenogenous-based human platelets were effective in the healing and regeneration of the bony tissue in experimentally induced bone defect model in rabbits, highlighting that the xenogenous platelets due to their availability may be a valuable option for tissue engineering purposes [29,35,39].

Given the above explanations, we produced a novel xenogenous-based bovine PG and embedded it within a xenogenous-based three-dimensional collagen implant (CI). We combined platelets with the scaffold to produce a bioactive graft, to increase the efficacy of the scaffold and to test whether the bovine PG in combination with the CI is an effective way to restore the structure and function of the neotenon in an experimentally induced large Achilles tendon defect model in rabbits. We hypothesized that:

1. The bovine PG may increase cell viability, proliferation, differentiation and matrix production *in vitro* because of its growth factors.
2. Due to its xenogeneic base and growth factors, it may increase inflammation at short term, which may be beneficial for tendon healing because such a short-term inflammatory response may enhance the fibroplasia and remodeling phases.
3. The bovine PG may enhance biocompatibility, biodegradability and incorporative properties of the implant.

## 2. Materials and methods

### 2.1 Ethics

All animals received humane care in compliance with the Guide for Care and use of Laboratory Animals published by the National Institutes of Health (NIH publication No. 85 – 23, revised 1985). The study was approved by the local Ethics Committee of our Veterinary School.

### 2.2 Preparation of the CI

Collagen type I was extracted from the bovine tendon, and its purity was confirmed by SDS–PAGE [13]. The acid-solubilized collagen molecules were electrospinned onto a dual plate device to produce the large and aligned electrospun collagen fibers [10]. After electrospinning, the acid-solubilized bovine tendon type I collagen molecules were mixed with the electrospun collagen fibers and polymerized in an incubator at 4°C for 48 h to produce the tridimensional collagen gel [3]. The collagen was aligned under 12 Tesla magnetic fields (CRETA, Grenoble) during polymerization. The collagen composite was cut into several pieces of the same size and shape as the rabbit’s Achilles apparatus. The collagen composites were crosslinked after suspension in iso-osmolar 0.1% riboflavin solution, using ultraviolet (UV) irradiation (365 nm wavelength) [14]. The final product was repeatedly washed with distilled water and received 100 Gray g-radiation and suspended in ethanol 96% to sterilize and maintain its sterility until surgery [9]. The morphology of the scaffold was studied by scanning electron microscopy (SEM). Sterility and endotoxin content were tested and confirmed by microbiological and limulus amebocyte lysate tests, respectively. The scaffolds were seeded by the Rat skin ﬁbroblasts (cell line CRL-1213), and the cell viability was determined and confirmed by histology, SEM and live/dead cell assay (Supplementary 1: Protocol S).

### 2.3 Preparation of the PG embedded within the CI

The blood was harvested from healthy bovines and transferred into the sterile EDTA tubes (1.5 mg/ml of blood). The animals were free of any contagious and particularly zoonotic diseases such as bovine spongiform encephalitis, rabies, tuberculosis and brucellosis, and the safety of the blood samples were tested and approved by a certified laboratory. The samples were spin at 1500 r.p.m. (215*g*) for 15 min. On centrifugation of anticoagulated blood, the following three layers were formed: RBCs (bottom), WBCs/platelets (buffy coat) (middle) and plasma (top). Under a laminar flow hood in a clean room, the plasma and buffy coat were suctioned into the new tubes and centrifuged again (1500 r.p.m.). The RBCs were discarded. Three layers including WBCs (bottom), PRP (middle) and platelet-poor plasma (PPP) (top) were formed. The PPP and PRP were suctioned into the new tubes and the WBCs were discarded. The pure platelets were harvested, and the purity of the solution was tested using light microscopy. For this purpose, three samples were fixed on the glass slides and stained with Wright–Giemsa staining; it was confirmed that the samples were free of bovine WBCs and RBCs. The platelets were counted with a standard hemocytometer, and the total platelet count was calculated for each sample [24,40]. After PRP-PPP preparation, the samples were lyophilized and transformed to powder. The powder was sterilized by UV irradiation, and the sterile powder was solved in the sterile phosphate buffered saline (0.9% NaCl). The final platelet concentration per each microliter was set to be 2,000,000 platelets/µl. This concentration was 6 – 7 times higher than the normal platelet concentration (300,000 platelets/µl) in blood circulation.

**Figure 1.**
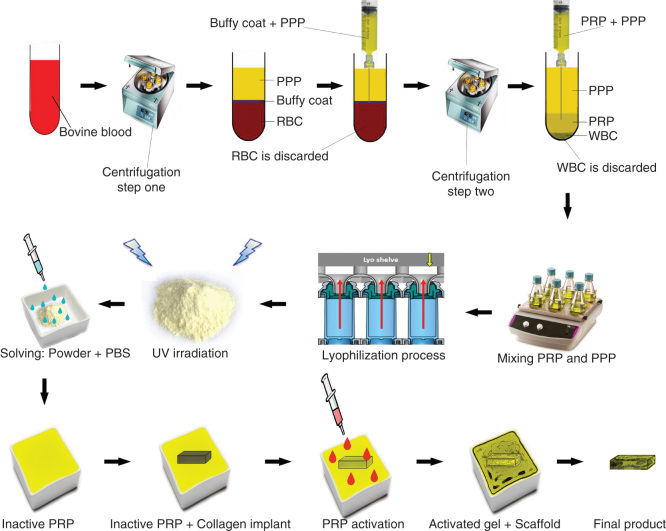
**Schematic view of the xenogenous-based bovine-pure PG preparation and its embedding with the CI.** Peripheral blood was obtained by i.v. catheterization of the healthy bovine and was transferred to the EDTA tubes. The samples were centrifuged in two steps. After the first-step centrifugation, three layers are formed including PPP, PRP + WBCs = buffy coat and RBCs. The PPP and buffy coat layers were suctioned and transferred into a new tube but the RBCs were discarded. The PPP and buffy coat were centrifuged again (second step centrifugation). This resulted in formation of three layers including PPP, PRP and WBC. WBCs were discarded, but the PPP and PRP were suctioned into the sterile tubes. The solution was mixed and lyophilized to concentrate the platelets and produce a lyophilized platelet powder. The powder was then sterilized by UV irradiation, and it was then solved in sterile phosphate buffer saline to produce the desired concentration (six to seven times more than that of the normal blood concentration). The sterile fully dehydrated three-dimensional CI was immersed in the PRP solution, and the implant was left to absorb the platelets in its architecture. The PRP solution was then activated, using bovine thrombin and CaCl_2_. Therefore, the absorbed PRP in the CI was activated and the gel was formed in the CI. The final product was a PG embedded within the CI. Each arrow shows a subsequent step.

**Figure 2.**
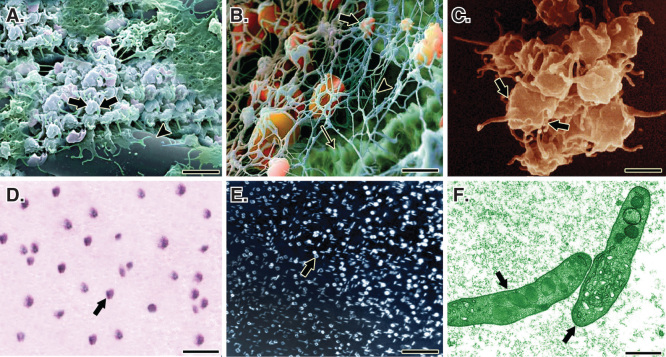
**A**. A SEM of the PG embedded within the CI. Note that the platelets (arrows) are well attached to the collagen fibers (arrow head) of the bioimplant. **B.** In a larger magnification, the activated platelets (thick arrow) are attached to the fibrin matrix (arrow head) and the collagen fibers of the implant (thin arrow). **C.** A SEM of the activated and accumulated platelets. **D.** Cytological figure of the bovine platelets after the second step centrifugation. Note that the cytological section is filled with only the platelets. **E.** The cytologic section of the bovine PRP under polarized microscope. **F.** The TEM of the bovine platelets after the second centrifugation. Scale bar: **A**, 1 µm; **B**, 750 nm; **C**, 500 nm; **D**, 3 µm; **E**, 12 µm; **F**, 630 nm.

CI: Collagen implant; PG: Platelet gel; SEM: Scanning electron microscopy; TEM: Transmission electron microscopy.

Two milliliters of PRP was transferred into a sterile self-costume made rectangular dish. The fully dehydrated CI was weighed and then placed in the dish. After 30 min, the scaffolds fully absorbed the solution. Two milliliters of the platelets was activated, using a combination of bovine thrombin (5000 U) and 5 ml of 10% CaCl with a proportion of 10 platelet solution: 1 activator [40]. This produced a PG embedded within the CI (Figure 1). The bioactive grafts were subjected to SEM, inverted microscopy and histology, and the presence of the platelets in the internal parts of the scaffold was confirmed (Figure 2).

CI: Collagen implant; i.v.: Intravenous; PG: Platelet gel; PPP: Platelet-poor plasma; PRP: Platelet-rich plasma; RBC: Red blood cell; WBC: White blood cells.

### 2.4 Live/dead cell assay and immunofluorescence microscopy

Rat skin ﬁbroblasts (cell line CRL-1213) were seeded on the collagen and collagen-platelet scaffolds (Supplementary 1). The scaffolds were grown *in vitro* in a 5% CO_2_ incubator at 37°C for 5, 10 and 20 days, with the medium (Dulbecco’s Modiﬁed Eagle Medium supplemented with 10% fetal bovine serum, 20 U/ml penicillin and 20 µg/ml streptomycin [Invitrogen, Carlsbad, CA, USA]) being replaced every 3 days. Cell–scaffold interaction was observed by SEM. Cell viability was determined by live/dead cell assay, using ﬂuorescein diacetate (live) (FDA, Molecular Probes, Invitrogen Corporation) and propidium iodide (dead) (Cayman Chemical Company, MI, USA). The scaﬀolds (n = 10 collagen; n = 10 collagen-platelets) with the ﬂuorescence stained cells were viewed under a Nikon ﬂuorescent microscope. Number of the live stained cells and dead stained cells were counted by the computer software (Image J, NIH, USA). The viability index was analyzed as: viability index = (number of viable cells/total number of cells) × 100 [3].

Cell morphology and cell–scaffold interaction were also studied by immunofluorescence microscopy. The scaffolds with cells on a 25-mm coverslip were washed twice with PBS. The cells were fixed in 4% paraformaldehyde, at room temperature (RT), for 60 min. The fixed cells were then washed twice with 0.02% PBS/sodium azide and permeabilized with 0.2% saponin for 10 min. The nonspecific sites were blocked by incubation in 0.02% PBS/1% bovine serum albumin (BSA)/0.02% sodium azide for 10 min at RT. The primary antibody (anti-Grp78, also known as BiP at a 1:100 dilution) was added to the coverslip to completely cover the surface and allowed to incubate at RT for 45 min. The coverslip was rinsed three times with 0.02% PBS/sodium azide and then blocked again with PBS/BSA/sodium azide for 10 min. The secondary antibody (Alexa 488 goat anti-rabbit at a 1:100 dilution) was added to the coverslip and incubated at RT for 45 min. The coverslip was rinsed three times with PBS/sodium azide and then incubated in 2 ml of a DAPI (4′,6-diamidino-2-phenylindole) solution of 0.5 ng/ml for 15 min. The coverslip was rinsed with PBS/sodium azide, then with deionized water and mounted on a glass slide with Fluoromount G.

### 2.5 Platelet growth factor level

The platelet-derived growth factor (PDGF) and IGF-I levels of the platelets derived after the first centrifugation (750,000 platelets/µl, n = 10 samples), the platelets derived after the second centrifugation (1500000 platelets/µl, n = 10 samples) and the platelet solution after platelet lyophilization and saline solving procedure (2,000,000 platelets/µl, n = 10 samples) and the PG (2,000,000 platelets/µl, n = 10 samples) were measured, using a commercially available Quantikine ELISA kits (DHD00 and DG100, respectively, R&D Systems, Minneapolis, MN, USA) [20]. To measure the PDGF-AB level, the samples and standards were prepared according to the manufacturer’s protocol. The samples were incubated for 2 h, washed and incubated with enzyme-conjugated antibodies directed against PDGF-AB for an additional 2 h at RT. The wells were then washed and the substrate was added 20 min at RT. The stop solution was added to each well, and the absorbance was determined at 450 nm, using a microtiter plate reader. For measuring IGF-I, a dilution series of IGF standards was prepared in 100 µl volumes in 96-well microtiter plates coated with a monoclonal antibody specific for IGF-I. The microtiter plate was incubated for 2 h at 2 – 8°C. The wells were washed three times and incubated with enzyme-conjugated IGF-I for 1 h at 2 – 8°C. The wells were washed three times again; the substrate solution was added and the plates were incubated for 30 min at RT. The stop solution was added to each well, and the absorbance for each was determined, at 450 nm, using a microtiter plate reader.

### 2.6 Platelet aggregation test

Platelet aggregation was tested by light transmission aggregometry (LTA) as previously described [41]. LTA measures the changes in transmission of a beam of light through a sample of PRP or platelet suspensions in buffer, which occur when platelets change shape and aggregate upon stimulation. In the LTA method (Chrono-log series 400, Harvertown, PA, USA), collagen (3.2 µg/ml) and TRAP-6 (32 µM) (Verum Diagnostica, Munich, DE) were used as agonists. The results were expressed as maximal light transmission. The LTA of the platelets derived after first centrifugation (750,000 platelets/µl, n = 10 samples), the platelets derived after second centrifugation (1,500,000 platelets/µl, n = 10 samples) and the platelet solution after platelet lyophilization and saline solving procedure (2,000,000 platelets/µl, n = 10 samples) were statistically compared.

### 2.7 Animals and grouping details

**Figure 3.**
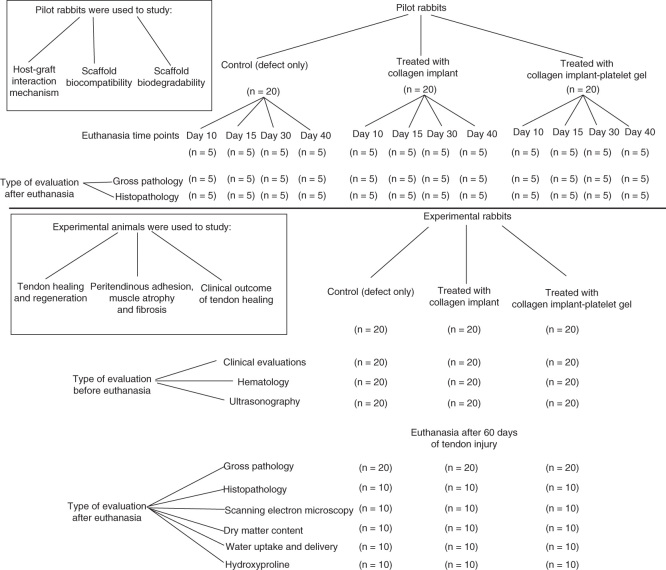
**Schematic description of grouping details and evaluation methods.**

Sixty skeletally mature male White New Zealand rabbits of 12 ± 2 month’s age and 3.41 ± 0.25 kg body weights were randomly selected for this experiment. In each animal, the left hindlimb was selected to make a large tendon defect and the right one was left intact. The animals were randomly distributed into three groups of control (defect without implant) (n *=* 20), treated with collagen implant (TCI) (n *=* 20) and treated with collagen-platelet gel (TCI-PG) implant (n *=* 20). Another 60 animals were used as a pilot model to determine the host–graft interaction mechanism and to evaluate the tendon healing process at earlier stages. The pilot animals were randomly assigned to three equal groups of control (n *=* 20), TCI (n *=* 20), TCI-PG (n *=* 20). Each group was then divided into four subgroups of 10 (n *=* 5), 15 (n *=* 5), 30 (n *=* 5) and 40 (n *=* 5) days post-injury (DPI) (Figure 3).

### 2.8 Premedication and anesthesia

Premedication was provided by intramuscular injection of 1 mg/kg Acepromazine maleate, and the animals were anesthetized by intramuscular injection of 15 mg/kg Ketamine +0.05 mg/kg Xylazine hydrochloride (All from Alfasan Co, Woerden, Netherlands) [6].

### 2.9 Injury induction and surgical reconstruction

The left hind limb of all the animals was prepared aseptically. After the longitudinal skin incision over the Achilles apparatus (calcaneal complex), 2 cm of the Achilles tendon (all the three strands) with the covering paratenon (the paratenon of all the three Achilles strands) were completely excised by transverse incisions, ∼ 5 mm distal to the gastrocnemius muscle and 5 mm proximal to the calcaneal tuberosity [10].

**Figure 4.**
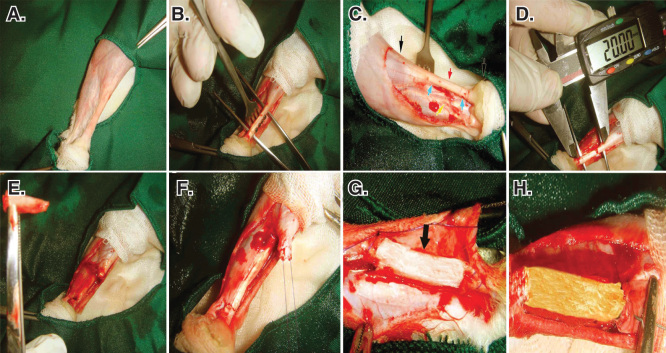
**Induction of tendon defect and surgical reconstruction.** **A.** The surgical site and preparation method. **B.** Skin incision and exposure of the Achilles apparatus. **C.** Black arrows show the gastroc-soleus muscle proximally and calcaneal tuberosity distally. The red arrow shows the Achilles apparatus, blue arrows show the segment to be removed and the yellow arrow shows the *tibialis* *posterior* tendon. **D** and **E.** Two centimeters of the Achilles tendon was measured with a digital caliper and removed. **F**. A modified Kessler Core pattern suture was anchored at the edges of the remaining tendon. **G** and **H**. The collagen and collagen-PG implant was inserted.

Primary reconstruction of the tendon extremities was conducted, using double-strand modified Kessler Core pattern (PDS 0-4, Ethicon, Inc., 1997, Johnson & Johnson, USA) [3]. This aligned the remaining tendon extremities in a normal anatomical position and created a 2-cm gap between the extremities [14]. The same method was applied to all the groups by the same surgeon. For insertion of the prosthetic implants in the tendon gap, a double-strand suture was passed through the longitudinal axis of the implant [7]. The subcutaneous fascia and the skin over the lesion were closed routinely (Figure 4). Postoperative analgesia with fentanyl (Matrifen, Roskilde, DK; 0.0015 mg/kg/h) was provided for 3 days via a transdermal patch applied to the depilated and sutured skin [3].

PG: Platelet gel.

### 2.10 Clinical examinations

Physical and behavioral statuses of the animals were monitored, weekly (n *=* 20 for each group). Tarsal flexion degree, weight distribution per limb, pain on palpation, heel and toe position and swelling of the injured area were weekly monitored and scored (Supplementary 2: Table S1) [3]. At weekly intervals, the transverse diameter (TD) and surface temperature (ST) of the injured and the contralateral areas (n *=* 20 for each group) were measured, using a micrometer measurement device (Guanglu electronic digital caliper, Anyang, South Korea) and a laser heat-detector device (Mastech, MS6530 Infrared Thermometer, Seoul, South Korea), respectively [3].

### 2.11 Hematology

At days 0 (before injury), 10 and 60 of tendon injury, 1-ml blood sample was collected from each animal (n *=* 20 for each experimental group), transferred into the EDTA tube and indirectly tested for different cell typing, using standard clinical cell-counter (Veterinary Auto-analyzer, Cambridge, UK) [6].

### 2.12 Ultrasonography

The injured and normal contralateral tendons (NCTs) (n *=* 20 for each group) of all the animals were evaluated by ultrasonography at weekly intervals. Transverse and longitudinal ultrasonographic images were obtained with a 7.5 – 12 MHz linear probe (Simense SLR-400, Berlin, Germany; Echo wave 3.23 software). Echogenicity, homogenicity, peritendinous adhesion, healing quality and regeneration volume were assessed and scored (Supplementary 2: Table S2) [13].

### 2.13 Euthanasia

The animals were anesthetized by intramuscular injection of a 15 mg/kg Ketamine, 2 mg/kg Xylazine and 1 mg/kg Acepromazine maleate (All from Alfasan Co). Then, they were euthanized by intracardiac injection of 1 mg/kg Gallamine triethiodide (Specia Co., Paris, France).

### 2.14 Sample collection

Immediately after euthanasia, each injured tendon and its contralateral intact one were dissected and assessed for gross pathology (n *=* 20*^L^* and 20*^R^* for each experimental group at each time point; n *=* 5*^L^* and 5*^R^* for the pilot groups at each time point). The samples from each experimental group were randomly divided into two equal subgroups. Subgroup 1 (n *=* 10*^L^*, 10*^R^*) was used for biophysical and biochemical testing and subgroup 2 (n *=* 10*^L^*, 10*^R^*) was used for morphological analyses. The tendons in subgroup 2 were longitudinally divided into two parts. Part A (medial part) was used for histopathology and part B (lateral part) was processed for SEM [9].

Lower than 3 min after euthanasia, the histologic and SEM samples were transferred to formalin and glutaraldehyde at the time of sampling. For dry matter content and physical properties-related methods, the investigation was immediately started after sampling. Biochemical samples were freezed at − 80°C. In that temperature no tissue degradation occurs. The freezed samples were used for biochemical analyses, 3 days later after sampling.

### 2.15 Gross pathology

Hyperemia, peritendinous adhesion, general appearance, muscle atrophy and fibrosis together with the tendon diameter were scored and measured (Supplementary 2: Table S3) [3].

### 2.16 Hydroxyproline

The hydroxyproline concentration was measured by spectrophotometry [6]. The samples were hydrolyzed in 6 M HCl at 105°C for 14 h, and the hydroxyproline was oxidized by chloramines T. Then by adding Ehrlich’s reagent and incubating at 60°C, a chromophore was formed. To remove the interfering chromophores, the hydroxyproline product in alkaline media was extracted into toluene and then into acid phase. The absorbance of acid phase was read at 543 nm, and the hydroxyproline content was calculated from the calibration curve based on the standard solutions run the same as the samples.

### 2.17 Determination of water uptake, water delivery and percentage dry weight (biophysical characteristics)

The percentage of dry matter content was calculated as: [dry weight (Wdry)/wet weight (Wwet)] × 100. To determine the water uptake capacity (WUC), each fully dehydrated sample (n *=* 10 left, n *=* 10 right for each group) was immersed in 0.9% saline (at 37^o^C) for 48 h to gain its final Wwet. This weight was used as an index of Wwet of the sample, which was then dried. The fully dehydrated sample was weighed and then immersed in 0.9% saline (at 37°C). From min 1 to 960 after immersion, the sample was weighed at various time points. The WUC was calculated as: WUC index = Wdry/time. The time is the point that the sample gained its maximum Wwet. For calculation of the water delivery capacity (WDC), the fully hydrated sample was placed in a dry place (at 37^o^C) and left to evaporate its water content. From min 1 to 7680 after air exposure, the sample was weighed at various time points. The WDC was calculated as: WDC index = Wwet/Wdry × 100/time. The time is the point that the sample gained its maximum Wdry.

### 2.18 Histopathologic and histomorphometric analyses

After fixation in 10% neutral buffered formalin, the tendon samples were washed, dehydrated in a graded series of ethanol, cleared in xylene, embedded in paraffin wax and longitudinally and transversely sectioned at 4 µm in thickness and stained by hematoxylin and eosin [7]. The samples were examined by a light microscope (Olympus, Tokyo, Japan). The photomicrographs were captured from the histologic fields and then transferred to the computer software (Adobe Photoshop CS-5, CA, USA) for digital analysis (n^sample^ = 10, n^histopathologic section^ = 5, n^histopathologic field^ = 5, totally 250^L^ and 250^R^ histopathologic fields for each group). Total cellularity, immature tenoblasts, mature tenoblasts, tenocytes, neutrophils, lymphocytes, macrophages, plasma cells and blood vessels of the tendon proper were counted (magnification ×200) [9]. The collagen mass density was analyzed by computer software (Image J, NIH, CA, USA) and reported as percentage (magnification ×200). Crimp pattern, tissue alignment and perivascular edema were scored (magnification ×200) (Supplementary 2: Table S4) [3].

### 2.19 Scanning electron microscopy

The samples were fixed in cold glutaraldehyde 2.5%, coated by hexa-methyl-desalysin (TAAB, Co., London, UK) and finally gold-coated. Under SEM (Cambridge, London, UK), cell number (×200), cell density (×200), diameter of the collagen fibrils (×30,000), collagen fibers (×10,000), collagen fiber bundles (×5000) and collagen density (×200) were measured in 100 ultramicrographs using SEM image analyzing software (Avizo Fire data visualization and analysis software, VSG Group. FEI Co. MA, USA; Image J, NIH, CA, USA; Adobe Photoshop CS-5, CA, USA) (Supplementary 2: Table S5) [3].

### 2.20 Statistical analysis

All the quantitative values were expressed as mean ± SD and significant differences of the measured values in each group were statistically tested, using paired-sample *t-*test. The significant differences of the measured values between multiple groups at one time point (e.g., 60 DPI) were tested using one-way ANOVA with subsequent Tukey’s post-hoc test and between multiple comparisons at multiple time points, repeated measures ANOVA was used. All scored values were expressed as median (min-max). Kruskal–Wallis *H* test was performed to analyze the scored values [3,9,10]. A p value of < 0.05 was considered statistically significant.

## 3. Results

### 3.1 Morphology of the collagen-platelet implant

Number of platelets/mm^3^ of the CI, as confirmed by histology, was 2 × 10^6^. At SEM, the platelets and fibrin were well distributed and infiltrated throughout the CI so that the implant homogenously absorbed the platelets. The structure of the platelets was confirmed by SEM and transmission electron microscopy. No significant differences were seen between the number of platelets in 10 different areas of the CI (p > 0.05) (Figure 2).

### 3.2 *In vitro* cell viability tests

**Figure 5.**
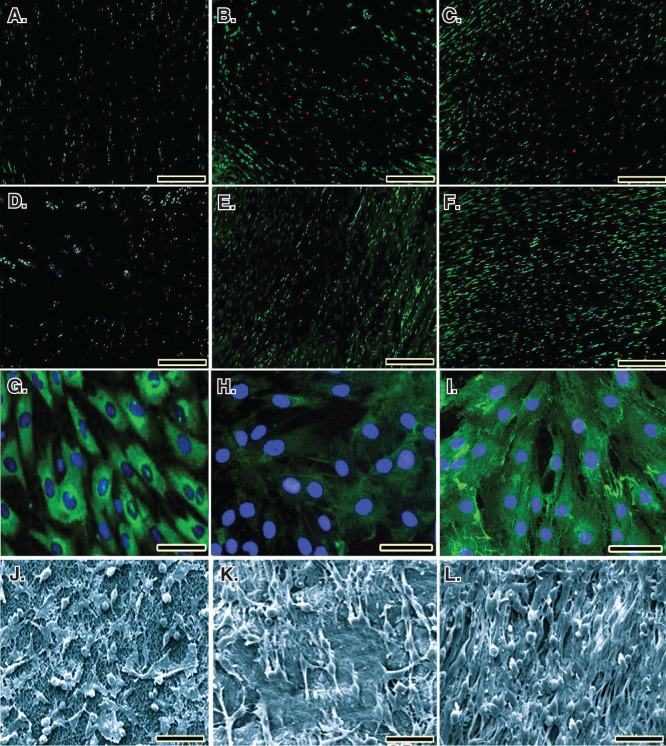
**Cell viability was determined by live/dead cell assay using ﬂuorescein diacetate (live) and propidium iodide (dead).** **A** to **C.** Rat fibroblasts that have been grown throughout the CI after 5, 10 and 20 days of cell seeding, respectively. **D** to **F.** Rat fibroblasts that have been grown throughout the collagen-platelet implant after 5, 10 and 20 days of cell seeding, respectively. The bovine xenogenous-based platelets in the collagen scaffold improved cell cytocompatibility of the implant because less dead cells (red) could be seen (**D** to **F**) when compared to the collagen scaffold alone (**A** to **C**). **G** to **I.** Fluorescence microscopy images of the fixed rat fibroblasts on culture medium, collagen scaffold and collagen-PG scaffolds, with rabbit anti-GRP 78 conjugated with Alexa 488 goat anti-rabbit (green)/DAPI (blue)-stained cells after 20 days of cell seeding and culture. **J** to **L.** SEM images of the rat fibroblasts cultured on culture medium, collagen scaffold and collagen-PG scaffolds after 20 days of cell seeding. Note that the PG enhanced cellular proliferation, maturation and matrix production. Scale bar: **A** to **F**: 100 µm, **G** to **I**: 25 µm and **J** to **L**: 50 µm.

CI: Collagen implant; DAPI: 4′,6-Diamidino-2-phenylindole; PG: Platelet gel.

In both the collagen (89.52%) and collagen-platelet (92.19%) implants, almost all the fibroblasts were green, indicating the cells were alive. Lack of propidium iodide (red) stained dead cells supports the idea that normal rat ﬁbroblasts were attached to the scaffold and that the majority of the cells were viable. Xenogenous-based bovine PG embedded within a CI significantly increased the cellular proliferation and cell viability (p < 0.05). Number of FDA-stained viable cells in the CI and CI-PG (× 200) were 127.82 ± 12.01^collagen-platelet^ versus 84.71 ± 9.41^collagen^ (day 5, p = 0.001), 274.82 ± 25.91^collagen-platelet^ versus 186.33 ± 27.91^collagen^ (day 10, p = 0.029) and 572.42 ± 57.77^collagen-platelet^ versus 321.34 ± 49.23^collagen^ (day 20, p = 0.001) and were 97.23^collagen-platelet^% versus 94.83^collagen^% (day 5), 95.32^collagen-platelet^% versus 92.16^collagen^% (day 10) and 92.19^collagen-platelet^ versus 89.52^collagen^% (day 20) of the total cellularity (vitality index) (Figure 4), respectively. After 5, 10 and 20 days of fibroblast seeding and culture into the CI and CI-PG, the qualitative results of the SEM and immunofluorescence microscopic studies showed that bovine platelets resulted in a better distribution of the cultured fibroblasts inside the scaffold and the cells were more mature and produced more collagenous matrix in the scaffold (Figure 5).

### 3.3 Level of the platelet growth factor

The PDGF-AB level of the platelets of the whole plasma and buffy coat derived after the first centrifugation (number of platelets = 750,000/µl), the platelets derived after the second step centrifugation (1,500,000 platelets/µl), the platelet solution prepared after lyophilization and saline solving procedures (20,000,000 platelets/µl) and the PG (activated platelets; 20,000,000 platelets/µl) were 74.18 ± 7.91, 243.27 ± 34.12, 169.88 ± 27.02 and 692.55 ± 102.38 ng/ml, respectively. The IGF-I level of the platelets of the whole plasma and buffy coat derived after the first centrifugation (number of platelets = 750,000/µl), the platelets derived after the second step centrifugation (15,000,000 platelets/µl), the platelet solution prepared after lyophilization and saline solving procedures (20,000,000 platelets/µl) and the PG (activated platelets; 20,000,000 platelets/µl) were 32.78 ± 12.32, 78.43 ± 8.19, 68.54 ± 10.92 and 132.47 ± 22.93 ng/ml, respectively.

### 3.4 Light transmission aggregometry

The LTA of the platelets of the whole plasma and buffy coat derived after the first centrifugation (number of platelets = 750,000/µl), the platelets derived after the second step centrifugation (1,500,000 platelets/µl), the platelet solution prepared after lyophilization and saline solving procedures (20,000,000 platelets/µl) were 78.34 ± 3.55, 74.36 ± 6.91 and 68.32 ± 5.92%, respectively. There were no significant differences between the LTA of platelets derived after the first step centrifugation and those derived after the second step centrifugation (p > 0.05). There were also no significant differences between the LTA of the platelets derived after the second step centrifugation and those prepared after lyophilization and saline solving procedures (p > 0.05).

### 3.5 Clinical findings

None of the animals died during the experiment, and all had good appetite and weight gain. Those animals TCI-PG significantly gained better scored values for the tarsal flexion degree of the injured limb, heel and toe position of the injured limb, weight distribution on the legs and pain on palpation compared to those TCI or left untreated (control, no implant) (p < 0.05) (Table 1).

**Table 1.** **Scored values.**

	**Group**	**Control (defect only) (1)**	**CI (2)**	**Collagen-platelet implant (3)**	**p value**	**p value**
	**Criteria**	**Median (min – max)**	**Median (min – max)**	**Median (min – max)**	**1 versus 3**	**2 versus 3**
Clinical scoring (sum scored values of 60 days)	Tarsal flexion degree of the injured limb	27.5 (23 – 31)	20 (18 – 24)	15 (10 – 18)	0.001	0.042
	Heel and toe position of the injured limb	19 (17 – 22)	14 (12 – 18)	9.5 (8 – 11)	0.001	0.001
	Weight distribution of the legs	29 (26 – 32)	22.5 (18 – 25)	17 (13 – 21)	0.001	0.048
	Pain on palpation	20 (18 – 24)	14 (12 – 17)	9.5 (7 – 15)	0.001	0.046
Ultrasonographical scoring (day 60 after injury)	Echogenicity	3 (2 – 3)	2 (1 – 3)	1 (0 – 1)	0.001	0.001
	Homogenicity	3 (3 – 3)	3 (1 – 3)	1.5 (0 – 2)	0.001	0.001
	Reduction in peritendinous adhesion	3 (2 – 3)	1.5 (0 – 3)	1 (0 – 2)	0.001	0.046
	Regeneration volume	5 (5 – 5)	2 (0 – 2)	1 (0 – 1)	0.001	0.033
	Regenerative proportion	4 (3 – 5)	2 (0 – 2)	0 (0 – 1)	0.001	0.049
Gross pathological scoring (day 60 after injury)	Peritendinous adhesion	3 (2 – 3)	2.5 (1 – 2)	1 (0 – 2)	0.001	0.036
	Hyperemia	3(2 – 3)	1.5 (2 – 3)	1 (0 – 1)	0.001	0.048
	Muscle fibrosis	3 (3 – 4)	3 (1 – 3)	1 (0 – 2)	0.001	0.044
	Muscle atrophy	3 (2 – 4)	3 (0 – 3)	1.5 (1 – 2)	0.001	0.027
	General appearance of the injured tendons	3 (1 – 4)	2 (0 – 3)	2 (0 – 2)	0.012	0.019
Histopathological scoring (day 60 after injury)	Collagen fiber alignment	3 (2 – 3)	1 (1 – 3)	0 (0 – 1)	0.001	0.042
	Perivascular edema	2 (2 – 3)	0.5 (0 – 1)	0 (0 – 1)	0.001	0.078
	Tissue maturity	3.5 (2 – 4)	1.5 (1 – 2)	0.5 (0 – 1)	0.001	0.001
	Crimp pattern	4 (4 – 4)	1.5 (1 – 2)	1 (0 – 2)	0.001	0.154
	Vascularity	4.5 (4 – 5)	1.5 (1 – 3)	0.5 (0 – 2)	0.001	0.016
SEM scoring 1(day 60 after injury)	Collagen fibril alignment	3.5 (3 – 4)	2.5 (1 – 3)	1 (0 – 2)	0.001	0.029
	Collagen maturity	4 (4 – 4)	3(3 – 5)	2 (1 – 2)	0.009	0.033

All scored values were expressed as median (min – max). Kruskal–Wallis *H* test was performed to analyze the scored values. p < 0.05 was considered statistically significant. CI: Collagen implant.

**Figure 6.**
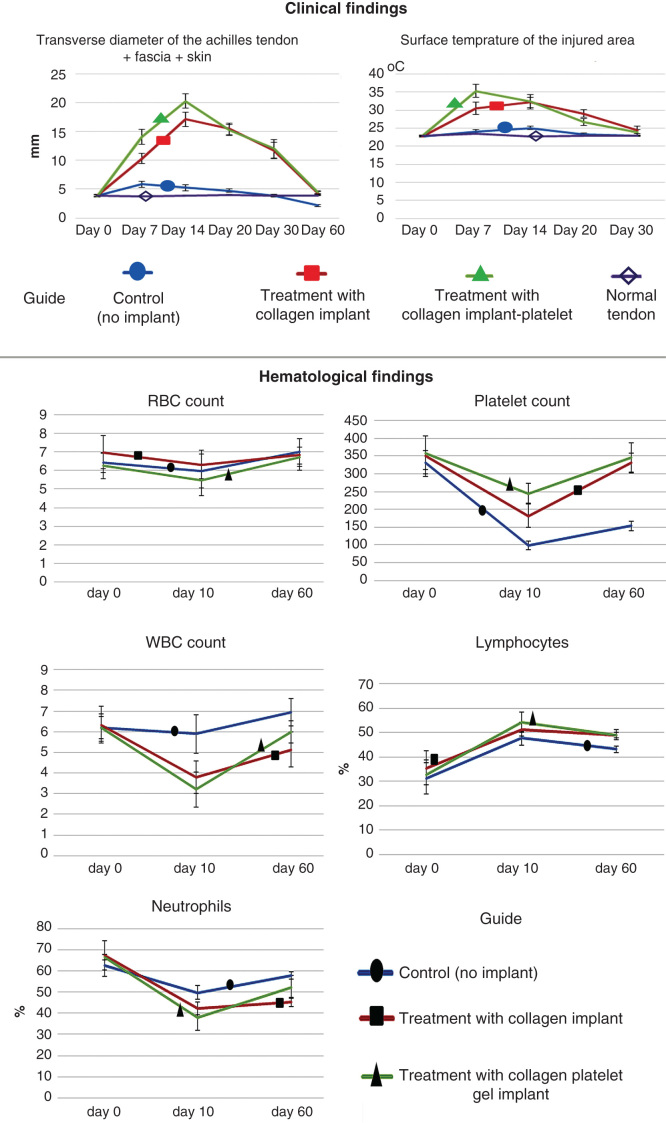
**Clinical measurements.** Note that the xenogenous-based bovine platelets embedded within the CI significantly increased the TD and ST of the injured area during the first 2 weeks after surgical operation as compared with the controls. These factors then gradually reduced to normal level up to 60 days of tendon injury. Hematological parameters of the injured animals: Note that surgical operation and implantation of the scaffolds did not alter the RBCs. The platelets decreased in all the groups after 10 days of surgical operation but unlike the controls, those animals treated with collagen-platelet implant showed normal platelet level at 60 days post-injury. Implantation of the collagen scaffold and collagen-platelet construct reduced WBCs in the blood of the treated animals compared to the control group at 10 days post-injury but the platelets returned to normal value at 60 days Post-injury. Ten days after tendon injury, the treated animals with collagen-platelet scaffold showed higher lymphocyte and lower neutrophil counts compared to the controls but at 60 days post-injury, the lymphocytes were still lower but the neutrophils increased to normal value.

CI: Collagen implant; ST: Surface temperature; TD: Transverse diameter.

Before injury induction (day 0), no significant differences existed in the TD of the injured area between all the groups; however, at 7 DPI, implantation of the CI and CI-PG significantly increased the TD of the injured area as compared to the normal value and the control (injured control tendons, ICTs) (p = 0.001 for both). At this stage, the injured treated tendons with collagen-platelet implant (ITTC-Ps) had significantly higher TD than the injured treated tendons with collagen implant (ITTCs) (p = 0.001). Compared to 7 DPI, at 14 DPI, the TD of the ICTs decreased (p > 0.05) but this factor significantly increased in the ITTCs and ITTC-Ps (p = 0.001 for both). At 14 DPI, the ITTC-Ps had significantly higher TD than the ITTCs (p = 0.001). At 20 – 60 DPI, the TD of all the groups gradually decreased so that at the end of the experiment (60 DPI), the TD of the ITTCs and ITTC-Ps reached their normal value and no significant differences were seen between the treated tendons and their NCTs (p > 0.05). At this stage, the TD of the ICTs, was significantly lower than the ITTCs, ITTC-Ps and the NCTs (p = 0.001 for all) (Figure 6).

Before injury (day 0), no significant differences were observed between the ST of the injured area of all the groups (p > 0.05). At 7 DPI, the CI and CI-PG significantly increased the ST of the injured area compared to the control and also when compared to day 0 (p = 0.001 for all). At this stage, the ST of the ITTC-Ps was significantly higher than those of the ITTCs (p = 0.001). At 14 DPI, the ST of the ITTC-Ps reduced but this factor increased in the ITTCs and ICTs; however, these changes were not statistically significant when compared to 7 DPI (p > 0.05 for all). At this stage, the ST of the ITTCs and ITTC-Ps showed no significant differences (p > 0.05) but when compared to the ICTs, they were significantly higher (p = 0.001). At 20 DPI, the ST of all the groups reduced compared to 14 DPI. Compared to the ITTCs, the ITTC-Ps had significantly lower ST at 20 DPI, which was still significantly higher than the ICTs and the normal value (p = 0.001 for all). The ICTs reached their normal ST at 20 DPI, but the ITTCs and ITTC-Ps reached their normal value at 30 DPI (Figure 6).

### 3.6 Hematological findings

Compared to 0 DPI, at 10 DPI, the RBC count slightly reduced and then gradually increased to normal value at 60 DPI; however, the differences were not statistically significant at all-time points (p > 0.05 for all). Compared to day 0 (before injury), at 10 DPI, number of platelets significantly reduced in all the groups (p = 0.001 for all). At this stage (10 DPI), the lowest platelet count was seen in the control group (no implant), whereas the highest level was seen in those animals treated with CI-PG. At this stage, although the CI-PG-treated animals had higher platelet count than those of the CI-treated ones; the differences were not statistically significant (p > 0.05). At this stage, those animals received either the CI or the CI-PG, had significantly higher platelet count than the control animals. Compared to 10 DPI, at 60 DPI, the platelet count of all the groups significantly increased (p < 0.05). When compared to 0 DPI (before injury), at 60 DPI, the platelet count reached its normal value in the treated animals so that no significant differences were seen between the measured values of days 0 and 60 (p > 0.05). By contrast, in the control animals, the platelet count was significantly lower at 60 DPI than the normal value (day 0) (p = 0.001).

No significant differences were seen in the WBC count and percentage of lymphocyte and neutrophils at 0 DPI. Compared to 0 DPI, treatment with either the CI or CI-PG significantly decreased WBC count and neutrophil percentage, at 10 DPI, but significantly increased the lymphocyte percentage (p < 0.05). However, in the control group, these factors did not show any significant differences with the normal values, at 10 DPI (p > 0.05). Compared to 10 DPI, at 60 DPI, WBC count and neutrophil percentage increased and lymphocyte percentage decreased in all the groups. Compared to the CI-treated animals, bovine platelets restored the WBC count and neutrophil percentage in the CI-PG-treated animals so that there were no significant differences between the CI-PG-treated animals and the control animals (p > 0.05). At 60 DPI, CI-treated animals showed significantly lower WBC and neutrophil count compared to day 0 or normal value (p = 0.001 for both) (Figure 6).

### 3.7 Ultrasonographical findings

**Figure 7.**
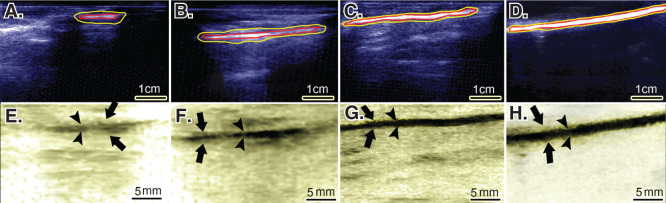
**Ultrasonographical characteristics of the injured and normal tendons. Longitudinal sections.** **A** to **D** are regular ultrasonographs, while **E** to **H** are inverted ultrasonographs. Red border (inner border) shows the tendon, while the yellow or outer outline shows the peritendinous adhesions in the healing tendon (**A** to **C**) and the paratenon in the normal tendon (**D**). Arrows head show the tendinous tissue within the defect area or the contralateral part (**E** to **H**), while the arrows show the peritendinous adhesions (**E** to **G**) and paratenon (**H**). In the ICTs (**A** and **E**), the healing regenerated tissue in the defect area shows hypoechogenicity, irregularity and an amputated echo texture pattern is seen around the tendon. Treatment with CI alone, improved the echogenicity and homogenicity of the healing tendon and also reduced peritendinous adhesion (**B** and **F**). Compared to the controls (defect and CI alone), treatment with collagen-platelet implant markedly improved the echogenicity, homogenicity and alignment of the newly developed tendon in the injured area and it also considerably decreased the peritendinous adhesions (**C** and **G**). Normal ultrasonographic pattern of the rabbit’s Achilles tendon is provided for comparison (**D** and **H**).

CI: Collagen implant; ICT: Injured control tendon.

Treatment with CI-PG significantly improved the scored values for the echogenicity, homogenicity, reduction in peritendinous adhesion, regeneration volume and regenerative proportion of the ITTC-Ps compared to the ITTCs and ICTs (p < 0.05) (Table 1; Figure 7).

### 3.8 Gross pathological findings

During different stages of tendon healing, no considerable healing response occurred in the ICTs, and the defect area was not filled with the fibrous connective tissue so that at the end of the experiment (60 DPI) only a loose areolar connective tissue that was tightly adhered to the surrounding fascia covered the defect area. The healing tendon was elongated when compared to the treated tendons. In the ICTs, the tendon edges were necrotized at the inflammatory stage of tendon healing, the gastroc-soleus muscle was atrophied and a vigorous muscle fibrosis was developed. The ICTs were hyperemic in nature and no tendinous tissue was formed. In contrast, the treated tendons showed a different healing pattern. Following implantation of the implants, a characteristic inflammatory response occurred around the implants. Compared to ITTCs, the ITTC-Ps showed more inflammatory response in the inflammatory phase of tendon healing. The ability of the implants to trigger the inflammation, produced a strong fibroplastic response in the defect area so that the implants were covered by the new granulation tissue soon after the tendon injury (e.g., after 10 days).

Although the inflammation was higher in the ITTC-Ps than the ITTCs, the implant was absorbed faster in the ITTC-Ps than the ITTCs. In the ITTC-Ps, the implant was replaced by a new tendon faster than the ITTCs. The ITTC-Ps had a more homogenous fibroelastic or tendinous surface especially at 60 DPI when compared to the ITTCs. Less muscle atrophy and fibrosis together with less peritendinous adhesions were observed in the treated lesions when compared to the control tendons.

**Figure 8.**
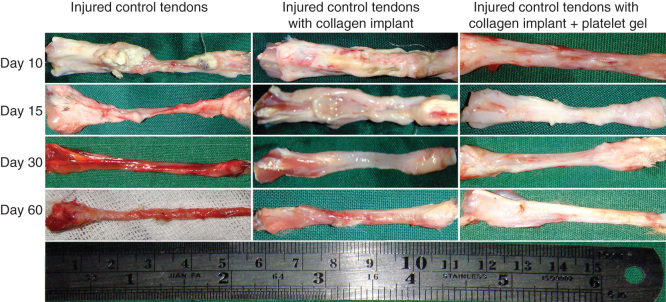
**Gross morphologic features of the injured tendons at various stages of tendon healing.** Note that no tendinous tissue has been formed in the ICTs (no implant), while a tendon has been formed in those groups treated with collagen and collagen-platelet scaffolds. Compared to the CI alone, treatment with the collagen-platelet implant considerably increased the rate of implant absorption and matrix replacement, it also improved the surface quality, fibrous density and reduced hyperemia and tendon adhesion to the surrounding structures. Bovine platelet also reduced muscle atrophy and fibrosis compared to the controls.

CI: Collagen implant; ICT: Injured control tendon.

At 60 DPI, the ICTs had smaller TD (short [S]: 1.19 ± 0.29 mm; large [L]: 2.24 ± 0.49 mm) than the ITTCs (S: 2.12 ± 0.25 mm; L: 3.31 ± 0.36 mm) and ITTC-Ps (S: 3.14 ± 0.33 mm; L: 3.91 ± 0.42 mm) (p = 0.001 for all). At this stage, the short TD of the ITTC-Ps was significantly greater than the ITTCs (p = 0.001). At this stage, the TD of the ITTC-Ps was comparable to the NCTs (S: 3.38 ± 0.27 mm; L: 3.96 ± 0.49 mm; p > 0.05). The ITTCs and ITTC-Ps showed significantly better scored values for the peritendinous adhesion, hyperemia, muscle fibrosis, muscle atrophy and general appearance of the injured tendons compared to the ICTs (p < 0.05) (Table 1; Figure 8).

### 3.9 Dry matter and hydroxyproline content

**Figure 9.**
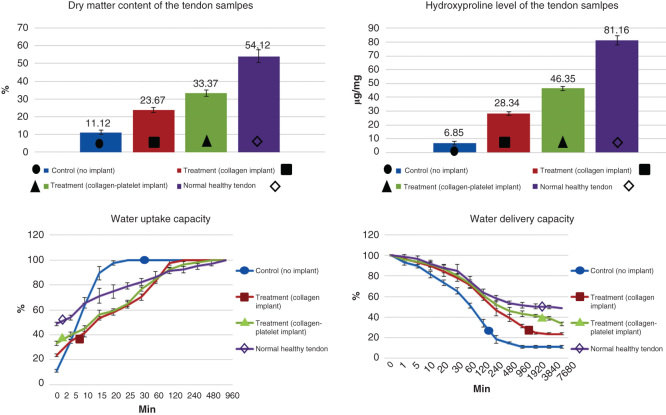
**Sixty days after tendon injury, bovine xenogenous-based platelets embedded within the CI significantly improved the dry matter and hydroxyproline content when compared with the controls (those treated with no implant and CI).** At this stage, those treated with collagen-platelet scaffold showed more similar pattern of water uptake and delivery to normal tendons compared to the controls (no implant and CI alone).

Both the ITTCs and ITTC-Ps showed significantly higher dry matter content and hydroxyproline level, at 60 DPI, compared to the ICTs (p = 0.001). In addition, the ITTC-Ps had significantly higher dry matter content and tissue hydroxyproline than the ITTCs at this stage (p = 0.001). Although the ITTC-Ps had significantly higher dry matter content and hydroxyproline compared to the controls, the measured values for the ITTC-Ps were still significantly lower than the NCTs (p = 0.001 for both) (Figure 9).

CI: Collagen implant

### 3.10 Water uptake and water delivery characteristics

After saline immersion of the fully dehydrated tendon samples, the ITTC-Ps and ITTCs showed a close pattern, approximately similar to the NCTs, so that they absorbed hydration slowly. The pattern of water uptake of the ITTC-Ps was slightly closer to the normal pattern than the ITTCs. In contrast, the ICTs absorbed hydration faster and reached their maximum Wwet sooner than the ITTCs, ITTC-Ps and NCTs. The ICTs, ITTCs, ITTC-Ps and NCTs reached their maximum Wwet after 20, 120, 480 and 960 min, respectively. At 60 DPI, the ITTC-Ps had significantly lower index of water uptake (0.069 ± 0.007) compared to the ICTs (0.556 ± 0.092) and ITTCs (0.197 ± 0.028) but this index was still significantly higher than the NCTs (0.050 ± 0.004; p = 0.001 for all).

After air exposure of the fully hydrated tendon samples, the ICTs reached their Wdry sooner than the ITTCs, ITTC-Ps and NCTs. The ITTCs also reached their Wdry earlier than the ITTC-Ps and NCTs. The pattern of water delivery of the ITTC-Ps was closer to the NCTs when compared to the ICTs and ITTCs. The ITTC-Ps and NCTs delivered their hydration slower than the controls. The ICTs, ITTCs, ITTC-Ps and NCTs reached their Wdry after 480, 1920, 7680 and 7680 min post air exposure of the fully hydrated samples, correspondingly. At 60 DPI, the ITTC-Ps (0.039 ± 0.005) gained significantly lower index of water delivery compared to the ICTs (0.936 ± 0.182) and ITTCs (0.110 ± 0.025) but this index was still significantly higher than the NCTs (0.026 ± 0.003) (Figure 9).

### 3.11 Histopathological findings

After tendon injury, the inflammatory cells such as neutrophils, macrophages and lymphocytes infiltrated in the defect area in all the groups. In the ICTs, although the inflammatory cells well infiltrated in the injured area, their number and density was low so that no well immune response occurred in these tendons. Due to this low inflammation, short term after injury, no well-quality granulation tissue consisting of mature well-differentiated fibroblasts and newly well-developed vascular structures formed midterm after injury. Thus, no well-characterized and strong fibroplastic response occurred in the ICTs, mid- to long term after injury. Finally, at 60 DPI, no sign of tendinous tissue formation was observed in the ICTs because the collagen fibers did not form in the injured area, the cells were immature and unorganized and no tissue alignment was evident in the defect area. Only a loose areolar connective tissue similar to the subcutaneous fascia consisting of many immature blood vessels and mesenchymal cells filled the defect area.

In contrast, implantation of the CI markedly increased the inflammatory response so that many neutrophils, lymphocytes and macrophages accumulated around the implant and gradually infiltrated in the inner parts of the implant. The immune behavior in response to collagen implantation, progressively degraded many areas of the CI. A good-quality granulation tissue was formed in the transition stage of the inflammatory stage to fibroplasia so that larger blood vessels regenerated and connected to the main blood vessels and more mature tenoblast were evident in the defect area. After the inflammatory phase diminished, some areas of the CI had been degraded but some remnants still preserved. The collagen remnants collaborated in tendon healing, but at short- to midterm, some remnants were surrounded by the chronic inflammatory cells and some of them were free of inflammatory cells accumulation. The fibroplastic response was strong in the ITTCs so that some collagen fibers formed along the direction of the collagen remnants. At remodeling phase, some degree of collagen alignment was evident and the proliferated cells showed some alignment in-line with the orientation of the collagen fibers. The collagen remnants were helpful in tendon formation because they acted as microscaffolds; however, they were mostly disappeared at long term due to degradation mechanism. Small portions were infiltrated by the host tenoblasts and accepted as a part of the neotenon.

**Figure 10.**
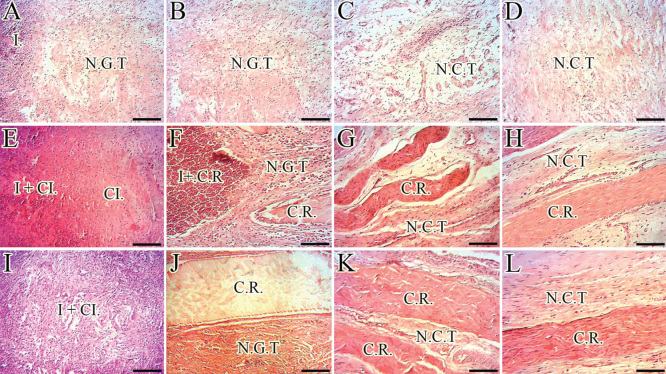
**Histopathologic features of the healing injured tendons at 10 – 40 days post-injury (DPI). **A** to **D** are ICTs, **E** to **H** are ITTCs, **I** to **L** are ITTC-Ps. **A**, **E** and **I** = 10 DPI; **B**, **F** and **J** = 15 DPI; **C**, **G** and **K** = 30 DPI; **D**, **H**, **L** = 40 DPI. Compared to the ICTs, implantation of the CI significantly increased migration and proliferation of the inflammatory cells in the injured area at 10 DPI (**E**). At 15 DPI (**F**), the inflammatory cells lysed some parts of the CI but these cells were still accumulated in some part of the CI. At 30 DPI, most parts of the CI degraded but few remnants incorporated with the newly developed connective tissue (N.C.T.). At 40 DPI, the preserved parts have been accepted as a part of tendon but the new tissue covering these remnants was still immature in nature (**H**). Compared to the ITTCs, treatment with platelets, considerably, improved the inflammatory cells infiltration so that the cells were well infiltrated all over the scaffold at 10 DPI (**I**), following no inflammatory cells accumulation at 15 DPI (**J**) so that the collagen remnants were free of inflammation. At 30 DPI, the collagen remnants (C.R.) were well accepted as parts of new tendon and a well-vascularized newly developed connective tissue regenerated between these remnants (**K**). At 40 DPI, the new connective tissue covering the accepted parts of the scaffold was considerably more mature than those seen in the ITTCs (**H**). Compared to the ICTs, the implants increased the inflammatory response at earlier stages of tendon healing which promoted a stronger fibrous response in the treated lesions. Scale bar **A** to **D**, **E**, **I** and **J** = 50 µm; **F**, **G**, **H** and **L** = 25 µm. Color staining = H&E.**

In contrast to the ICTs and ITTCs, implantation of the CI-PG in the defect area markedly increased the inflammatory response in the ITTC-Ps so that more inflammatory cells infiltrated in the implant but unlike the ITTCs, they did not accumulate in particular areas but well distributed throughout the implant and the defect area. This resulted in a better granulation tissue formation so that the ITTC-Ps had larger blood vessels, more mature tenoblasts and well-organized tissue at short term. Some parts of the implant degraded by the phagocytic cells. However, those parts, which were preserved midterm after injury, were not infiltrated by the inflammatory cells and properly collaborated at fibroplasia. A well-organized fibrous connective tissue regenerated between the preserved parts of the implant, thus the newly regenerated connective tissue aligned and matured along the direction of these preserved microscaffolds. Most of the implant remnants were gradually accepted as a part of the neotenon but fewer remnants gradually lysed and were absorbed by the enzymatic activity of the inflammatory cells. Long term after injury, a high-quality tissue replaced the implant in the defect area, which had comparable characteristics to normal tendon because its collagen fibers were well developed and properly aligned unidirectionally along the longitudinal direction, the cells were mostly tenocytes and mature tenoblasts and the collagen density was high (Figure 10).

CI: Collagen implant; C.R.: Collagen remnant; H&E: Hematoxylin and eosin; I.: Inflammation; ICT: Injured control tendon; ITTCs: Injured treated tendons with collagen implant; N.C.T: New connective tissue; N.G.T: New granulation tissue.

**Figure 11.**
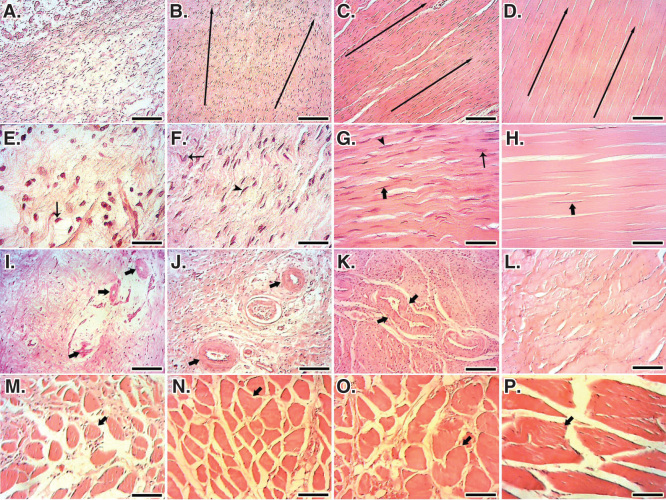
**Histopathology sections of the injured and normal tendons at 60 days post-injury (DPI).** **A** to **D.** Longitudinal sections of the tendons, scale bar = 50 µm. **E** to **H.** Longitudinal sections of tendons, scale bar = 12.5 µm. **I** to **L.** Transverse sections of the tendons, scale bar = 50 µm. **M** to **P**. Transverse sections of gastroc-soleus muscle, scale bar = 50 µm. **A**, **E**, **I** and **M** are ICTs (no implant); **B**, **F**, **J** and **N** are injured treated tendons with collagen implant (ITTCs); **C**, **G**, **K** and **O** are ITTC-Ps and **D**, **H**, **L** and **P** are normal contralateral healthy tendons (NCTs), for comparison. At 60 DPI, no tendinous tissue was formed in the ICTs (**A**) so that the density of the collagenous matrix was low (**A**) and the collagen-like fibers were randomly orientated at different directions (**A**). The cells in these tendons were immature tenoblasts (**E**, thin arrow) and no signs of cell alignment was seen (**E**). The newly developed vessels in these tendons were small and immature (**I**, arrows) and the muscle fibrosis and atrophy was obvious in this group (**M**, arrow shows a muscle fiber). In contrast to the ICTs, the collagen fibers formed in an aligned manner in the ITTCs and ITTC-Ps, (**B** and **C**, long arrows show the direction of the collagen fibers), the cells were mostly mature tenoblasts (arrow head) and tenocytes (thick arrow) and larger and mature vessels were regenerated in the injured area (**J** and **K**, arrows). The muscle fibrosis and atrophy were less than ICTs (**N** and **O**, arrows show muscle fibers). Compared to the ITTCs, the ITTC-Ps showed higher degree of collagen and cellular maturation and alignment, larger and thicker vessels and less muscle fibrosis and atrophy. Color staining = H&E.

At 60 DPI, the ITTC-Ps had significantly lower total cellularity, immature tenoblast and lymphocytes and had higher mature tenoblasts, tenocytes, large-sized blood vessels and collagen density, than the ICTs (p = 0.001). In addition, the ITTC-Ps had significantly higher total cellularity, more plasma cells, macrophages, neutrophils, number and diameter of blood vessels, and collagen and cell density than the ITTCs, at 60 DPI (p = 0.001) (Table 2). The ITTC-Ps and ITTCs showed significantly better scored values for the collagen fiber alignment, perivascular edema, tissue maturity, crimp pattern and vascularity compared to the ICTs (p < 0.05) (Table 1; Figure 11). Less muscle fibrosis and atrophy were observed in the treated lesions compared to the ICTs, at 60 DPI (Figure 11).

**Table 2.** **Histopathologic characteristics of the injured healing tendons after 60 days of tendon injury and surgical operation.**

	**Groups**			**p value**		
	**Control (no implant) (1)**	**Collagen (2)**	**Collagen-platelet (3)**	**1 versus 2**	**1 versus 3**	**2 versus 3**
Total cellularity (n)	523.41 ± 19.14	373.44 ± 8.01	428.64 ± 7.44	0.001	0.001	0.001
Total tenoblast and tenocyte (n)	334.24 ± 10.9	257.06 ± 5.31	280.21 ± 5.33	0.001	0.001	0.003
Immature tenoblast (n)	221.82 ± 10.83	96.41 ± 5.63	49.10 ± 5.96	0.001	0.001	0.001
Mature tenoblast (n)	111.44 ± 6.43	144.68 ± 8.99	195.60 ± 12.33	0.001	0.001	0.001
Tenocyte (n)	3.72 ± 1.64	17.15 ± 2.64	44.56 ± 3.05	0.001	0.001	0.001
Neutrophil (n)	7.29 ± 1.73	14.18 ± 2.04	29.31 ± 4.75	0.001	0.001	0.001
Lymphocyte (n)	87.31 ± 7.4	51.16 ± 4.3	59.58 ± 6.59	0.001	0.001	0.241
Plasma cell (n)	4.19 ± 1.54	2.67 ± 1.3	10.19 ± 1.37	0.102	0.001	0.001
Macrophage (n)	59.32 ± 8.19	43.23 ± 3.89	56.3 ± 2.54	0.026	0.491	0.001
Vessels (n)	12.35 ± 2.96	33.44 ± 3.93	51.85 ± 4.36	0.001	0.001	0.001
Small vessels (n)	7.84 ± 0.63	26.7 ± 2.02	15.48 ± 1.45	0.001	0.001	0.001
Medium vessels (n)	3.07 ± 0.88	3.67 ± 0.7	16.22 ± 1.45	0.791	0.001	0.001
Large vessels (n)	0.62 ± 0.56	3.09 ± 0.98	12.52 ± 1.93	0.001	0.001	0.001
Immature tenoblast (d) (µm)	7.93 ± 1.23	6.72 ± 0.87	5.06 ± 0.61	0.388	0.001	0.042
Mature tenoblast (d) (µm)	3.7 ± 0.15	3.35 ± 0.18	2.76 ± 0.37	0.046	0.001	0.021
Tenocyte (d) (µm)	1.69 ± 0.04	1.32 ± 0.04	1.2 ± 0.02	0.001	0.001	0.001
Small vessels (d) (µm)	11.9 ± 0.86	15.64 ± 1.03	24.43 ± 1.98	0.001	0.001	0.001
Medium vessels (d) (µm)	34.41 ± 1.58	42.9 ± 2.65	54.18 ± 2.13	0.001	0.001	0.001
Large vessels (d) (µm)	69.19 ± 4.08	82.47 ± 3.68	119.01 ± 5.25	0.001	0.001	0.001
Collagen density per histologic field (%)	8.21 ± 1.86	35.19 ± 3.3	60.52 ± 3.32	0.001	0.001	0.001
Cell density per histologic field (%)	24.77 ± 3.4	15.81 ± 0.98	21.32 ± 2.42	0.001	0.519	0.001
Background density per histologic field (%)	63.16 ± 2.27	49.63 ± 2.98	15.98 ± 1.24	0.001	0.001	0.001

One-way ANOVA with its subsequent post-hoc Tukey test was used to compare the significant differences between groups. p < 0.05 was considered statistically significant. Number of tendon samples in each group = 10, number of histologic tissue section in each tendon = 5 and number of histologic field in each tissue section = 5. Totally, 250 histologic fields were used to evaluate the histologic characteristics of the injured healing tendons. d: Diameter; n: Number.

### 3.12 Scanning electron microscopic findings

**Figure 12.**
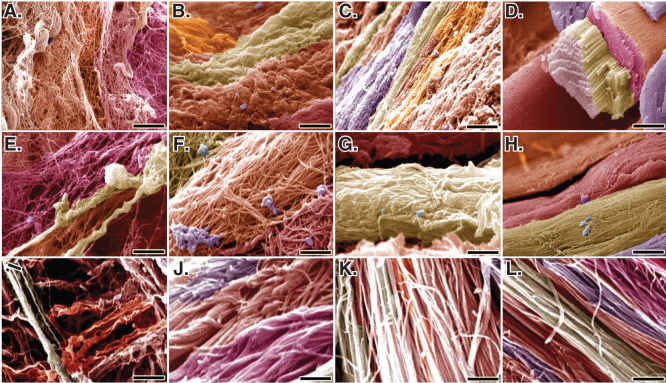
**SEM characteristics of the injured and normal tendons at 60 days post-injury.** **A**, **E** and **I** are injured control (no implant) tendons (ICTs). **B**, **F** and **J** are injured treated tendons with collagen implant (ITTCs). **C**, **G** and **K** are ITTC-Ps. **D**, **H** and **L** are normal healthy tendons for comparison. **A** – **H** are SEM figures at fiber level, while **I** to **L** are the SEM ultramicrographs at fibrillar level. Compared to the ICTs, those tendons treated with implants showed some degrees of collagen fibrils to fiber differentiation and fibrillar and fiber alignment. Compared to the ITTCs, treatment with platelets considerably improved collagen fiber formation (**G** vs **F**), collagen density and fiber and fibril alignment in the ITTC-Ps. Scale bar: **A** to **D** = 50 µm; **E** – **H** = 25 µm and **I** – **L** = 5 µm.

ITTCs: Injured treated tendons with collagen implant; ITTC-P: Injured treated tendons with collagen-platelet implant; SEM: Scanning electron microscopy.

At SEM level, bovine platelets improved the remodeling characteristics of the ITTC-Ps, so that the collagen fibrils properly filled the defect area and demonstrated an aligned and properly organized hierarchical organization in these tendons. The collagen fibrils of the ITTC-Ps were longitudinally connected to each other so that they were elongated between the muscle and bone. These fibrils were transversely aggregated and differentiated to collagen fibers so that the collagen fibers were morphologically well distinguishable at SEM level at 60 DPI. In contrast to the CI-PG, although the CI increased the collagen fibril density compared to the ICTs, these fibrils were of small-sized and showed lower alignment so that they haphazardly distributed in the healing area and the collagen fibers were not comparable to the normal tendon fibers. In the ICTs, actually no characteristic collagen fiber was diagnosed because the collagen fibrils were randomly oriented at different directions. In addition, their transverse and longitudinal diameters were low so that they had no longitudinal and transverse fibrils’ interconnection and their density was low; for this reason, the porosity (free spaces in the matrix) in the ICTs was high. This reflects the formation of a loose areolar connective tissue in the injured area. The posterior tibialis vessels and the regional nerves, which had been removed during the surgery because of their strict adherence to the Achilles apparatus, were also regenerated along the new ITTC-Ps. At 60 DPI, the ITTC-Ps had significantly higher TD of the collagen fibrils and fibers and the collagen fibrils’ density than the ITTCs and ICTs (p = 0.001 for all) (Table 3). In addition, the ITTC-Ps had significantly lower cellularity than the ICTs but had higher cellularity than the ITTCs at 60 DPI (p = 0.001 for both). Compared to the ICTs, the ITTCs and ITTC-Ps showed significantly better scored values for the collagen fibrils’ alignment and collagen maturity compared to the ICTs (p = 0.001) (Table 1; Figure 12).

**Table 3.** **SEM characteristics of the injured healing tendons after 60 days of tendon injury and surgical operation.**

	**Control (1)**	**Collagen (2)**	**PRP (3)**	**p value**		
				**1 versus 2**	**1 versus 3**	**2 versus 3**
TD of collagen fibrils (nm)	29.52 ± 3.27	46.21 ± 4.15	64.45 ± 4.92	0.001	0.001	0.001
TD of collagen fibers (nm)	-	4561.84 ± 147.46	6147.35 ± 277.32	0.001	0.001	0.001
Number of cells/per SEM field	257.33 ± 11.63	187.54 ± 7.55	208.17 ± 7.5	0.001	0.001	0.001
Density of the collagen fibrils per SEM field (%)	33.24 ± 4.43	58.88 ± 3.81	71.85 ± 3.57	0.001	0.001	0.001
Density of the cells per SEM field (%)	22 ± 3.3	18.86 ± 1.65	12.52 ± 1.11	0.192	0.001	0.001

One-Way ANOVA with its subsequent post-hoc Tukey test was used to compare the significant differences between groups. p < 0.05 was considered statistically significant. Number of tendon samples in each group = 10 and number of SEM fields in each tendon sample = 10. Totally, 100 SEM fields were used to evaluate the morphologic characteristics of each group. SEM: Scanning electron microscopy; TD: Transverse diameter.

## 4. Discussion

This is the first to report the role of bovine pure PG embedded within the CI on rabbit Achilles tendon healing. This study showed: i) bovine PG improved cell viability, proliferation, differentiation and matrix production *in vitro* and *in vivo*; ii) it increased inflammation short term after injury but did not prolong it. This resulted in an enhanced fibroplastic response; iii) in addition, the bovine PG improved the biocompatibility, biodegradability and healing incorporative properties of the CI, which then resulted in a better tenoinduction, tenoconduction, tenointegration and tendogenesis; and iv) finally, it produced a remodeled tendon that had superior structure and function than the controls.

An ideal tissue engineered graft should be cytocompatible *in vitro* to ensure that it has no cytotoxicity [8,13]. We showed that addition of the bovine platelets into the CI could increase the cytocompatibility of the CI, *in vitro*, so that an enhanced cellular proliferation and distribution in the CI-PG were observed compared to the CI. When the CI-PG inserted in the defect area, the bovine platelets motivated the inflammatory response sooner than the CI. Harris *et al*. [42] showed that PRP can initiate an inflammatory response in the absence of an inciting injury in normal soft tissues in rabbits.

At earlier stages of tendon healing, when the inflammation started, neutrophils had homogenously infiltrated throughout the injured area of the ITTC-Ps. In contrast, in the ITTCs, the neutrophils produced a demarcation line between the regenerated tissue and the CI. The neutrophils covered the peripheral area of the CI and few inflammatory cells successfully infiltrated in the inner parts of the CI. At later stages of inflammation (e.g., 10 – 15 DPI), the neutrophils’ count reduced and these cells were substituted by the chronic inflammatory cells (e.g., macrophages and lymphocytes). Number of macrophages was higher in the ITTC-Ps compared to the ITTCs.

Lymphocytes are important in tendon healing because they have a significant role in triggering the inflammation. They can handle the healing response to be more remodeling reaction rather than inflammatory reaction [4]. Lymphocytes do this mechanism by regulating the macrophage behavior [14]. Briefly, T-helper lymphocyte type I regulates the macrophage type I [3]. Macrophage type I is mostly a phagocytic cell, which could result in rejection of the implant [3]. T-helper lymphocyte type II regulates the macrophage type II, which has less phagocytic activity but can promote remodeling reaction; thus, its activation results in remodeling reaction which is equivalent to graft acceptance [6]. Both the CI and CI-PG triggered the number of lymphocyte and macrophages in the treated lesions compared to the ICTs at inflammation. This effect has been previously shown for both the collagen and platelets [42-44]. These inflammatory cells were infiltrated from the peripheral blood during tendon healing. This outcome is a constitution of healing process and has no relationship with the presence of WBCs in the PG [4,17].

Regarding the role of lymphocytes in graft acceptance or rejection and based on the various time points follow-up of the implant degradation and neotenon regeneration, it could be emphasized that the CI probably triggered the T-helper lymphocytes type II and the bovine platelets improved this beneficial effect. Macrophages have more role than the lymphocytes and neutrophils in tendon healing because they can modulate cell behavior by releasing several growth factors and cell stimulators but also similar to the neutrophils, they can phagocyte the scaffold remnants especially when the composition of the graft is a natural biodegradable molecule such as collagen [6,7,13]. Our morphologic evaluations at inflammation suggest that both the neutrophils and macrophages engaged with the implant degradation and the platelets controlled the phagocytic activity of these cells which may be due to the released growth factors of the platelets throughout the CI. Compared to the ITTCs, lower neutrophils and macrophage aggregation was seen around the collagen remnants and generally the inflammatory cells had better infiltration in the ITTC-Ps so that they were diffusely distributed all over the implant. It has been suggested that platelets have some growth factors that can modulate the inflammation but also can trigger cell behavior *in vitro* and *in vivo* [19,42,44].

Platelets resulted in a stronger inflammatory response when combined with the CI. Increase in the TD and ST of the injured area, presence of higher inflammatory cells throughout the injured area and decrease in the number of WBCs in the peripheral blood of the animals, all had correlations with each other, and are indices of inflammation and suggest a superior inflammatory response that had occurred in the ITTC-Ps when compared to the ITTCs and ICTs [6].

The role of inflammation and particularly macrophages in tendon healing should be highlighted because when activated they can produce a better fibroplastic response [4,12]. Macrophages have dominant role in transition of inflammation to fibroplasia because they modulate cell behavior and control their matrix production which has strong role in the quality of fibroplasia [4,10,14]. Although the platelets increased the inflammation at short term, the inflammation was only present at the first 2 weeks following injury and was not extended in the later stages of healing, suggesting the implants are biocompatible *in vivo*, and the bovine platelets have no hazardous effect on tendon healing.

Although the inflammatory cells were predominant at inflammatory stage, the platelets increased the healing capability of the CI, because the fibrous connective tissue, regenerated at the terminal stage of inflammation and fibroplasia, had started earlier in the ITTC-Ps than the ITTCs. We observed several migrating and proliferating mesenchymal cells including endothelial cells and immature tenoblasts in the ITTC-Ps at later stages of inflammation. In line with our findings, it has been shown that platelet-released growth factors promote the proliferation and maturation of tenocytes [18]. In a transected Achilles tendon tear model in rabbits, Lyras *et al*. [21] showed that PRP increases TGF-β1 expression during tendon healing. Also, Lyras *et al*. [45] showed that single injection of PRP in a ruptured Achilles tendon tear model in rabbits, significantly increases the IGF-I in the healing tendon.

Our morphologic observations at various time points suggest that the bovine platelets resulted in a more advanced fibroplasia in the ITTC-Ps compared to the ITTCs and ICTs because the newly regenerated tissue in the defect area of the ITTC-Ps was more homogenous, had several tenoblasts and blood vessels and a considerable metabolic activity, which we concluded this could be due to the presence of a higher collagenous matrix was produced by these cells at this stage (e.g., 15 – 40 DPI). Bovine platelets caused more tenoblast infiltration in the preserved collagen remnants thus improved the acceptability of the scaffold remnants. In addition, the platelets increased the degradation rate of some other areas of the scaffold and resulted in an enhanced tissue regeneration in the injured area so that most of the scaffold was absorbed or accepted at fibroplasia in the ITTC-Ps, but a considerable amount of scaffold remnants were still present in the ITTCs.

We selected 60 DPI for our main investigation because we believed that a better judgment could be made at remodeling phase of tendon healing [17]. At this phase, no tendinous tissue was formed in the ICTs, highlighting that the unassisted tendon healing could not repair such large tendon defects.

CI was effective in producing a neotenon in the ITTCs, but the characteristics of the ITTCs was far from and inferior to the ITTC-Ps. We showed that remodeling was superior in the ITTC-Ps compared to the controls. The higher transverse and longitudinal collagen fibrils’ diameter and density, together with the higher number of mature tenoblast and tenocyte, which were regenerated, distributed and oriented along the longitudinal axis of tendon, formation of collagen fibers from the new collagen fibrils and finally the presence of the more large-sized blood vessels in the ITTC-Ps, could well explain that the platelets were able to increase the quality and rate of tendon healing in a rabbit model [4,46].

The beneficial angiogenic effect of the platelets has possibly established a better circulation in the healing tissue. Lyras *et al*. [33] studied the effect of PG on angiogenesis in a transected model of rabbit Achilles tendon. They showed that there was significantly more angiogenesis in the PG group compared to the controls during the first 2 weeks of the healing process, that is, inflammatory and proliferative phases. Also, Vogrin *et al*. [22] showed that local application of PG enhances early revascularization of grafts in the osteoligamentous interface zone after anterior cruciate ligament reconstruction.

The above morphological results were confirmed by determination of the dry matter content, hydroxyproline level, water uptake and delivery. Dry matter content and hydroxyproline level of a tissue are indices of collagen content and correlate with the collagen mass density observed in the morphological assessments [3,14]. Water uptake and delivery of a tissue are indices of matrix organization and remodeling that correlate with the formation of the collagen fibers from new collagen fibrils and their alignment [3]. Based on our results, a normal tendon absorbs water slowly and delivers it slowly. The ITTC-Ps and ITTCs showed closer pattern to normal when compared to the ICTs. Also, the ITTC-Ps showed superior pattern of uptake and delivery compared to the ITTCs. These two characteristics have strong positive correlation with the biomechanical properties and also the dry matter and hydroxyproline contents of a tissue [3,7,10]. In line with our findings, Kaux *et al*. [47] showed that an injection of PRP in a sectioned rat Achilles tendon model influences the early phase of tendon healing and results in an ultimately stronger mechanical resistance.

In fact, the diameter, density, alignment and differentiation of the collagen fibrils and fibers have strong roles in tendon mechanical properties [3,15,46]. With improvement in these characteristics, a superior mechanical strength could be expected, which is responsible for weight-bearing capacity of the injured limb [4,9]. In addition, the combination of CI with bovine platelets reduced muscle atrophy and fibrosis and more importantly, decreased peritendinous adhesion formation at the wound site. All these three beneficial effects have strong clinical relevance [17].

Based on the hematologic and histopathologic results, the platelets enhanced chemotactical absorbance of the blood WBCs. Infiltration of these cells in the injured area produced a strong healing response. The inflammatory cells in the injured area together with the platelets and collagen fibers of the implant triggered migration of the mesenchymal cells into the injured area and prevented their randomized migration in the peripheral area of tendon [10]. Therefore, the healing response was concentrated in the defect area so that presence of higher tenoblasts in the injured area produced more collagen fibers in the defect area and infiltration of fewer tenoblasts on the peripheral area of tendon produced lower collagen fibers in the peritendinous area and this resulted in reduced adhesion formation around the treated lesions. Formation of a superior neotenon in the ITTC-Ps together with the lower adhesion formation resulted in an improved physical activity and weight-bearing in the treated animals; thus, lower muscle atrophy was observed in those animals treated with platelets compared with the controls. Correlations between the muscle fibrosis and atrophy and peritendinous adhesions with the reduced physical activity have been previously shown [3,14].

Although bovine platelets were xenogenous and increased the inflammatory response at short term, the outcome of this novel treatment strategy was excellent enough to ensure us about its efficacy. In fact, all the superiority of this treatment strategy was significant when compared to the controls. Although this study showed that rejection of xenografts may be a concern in clinical practice, this did not happen in the present study. We did not use the platelets with blood WBCs and only the pure bovine platelets were used. Dragoo *et al*. [44] evaluated the inflammatory effect of two different commercially available PRP systems, Biomet GPS III leukocyte-rich PRP (LR-PRP) versus MTF Cascade leukocyte-poor PRP (LP-PRP), after intra-tendinous injection in an animal model. They indicated that compared with the LP-PRP, the LR-PRP initiated a significantly greater acute inflammatory response at 5 days after injection. Perhaps, if we used bovine WBCs, the inflammation may be exaggerated which is not beneficial for tendon healing [17]. Recently, Kaux *et al*. [48] reported a case of exuberant inflammatory reaction after one injection of PRP to treat jumper’s knee in a 35-year-old male type 1 diabetic patient.

Combination of the platelets with the CI increased the tenoinductivity, because it increased cellular migration and proliferation in the injured area, enhanced the tenogenesis, because it increased the maturation and differentiation of the cellular and matrix components (collagen) to normal tendon cells and fibers, improved the tenoconduction, because it guided regeneration of the neotenon along the direction of the muscle to bone (normal anatomic direction of the tendon) and promoted tenointegration, because it increased incorporation of the graft and graft acceptability with the newly regenerating tissue.

Although bovine platelets were effective in tendon healing, it may be possible to increase their effectiveness by combining such strategy with rehabilitation programs and exercise activities, which has high value in sports medicine. Kaux *et al*. [49] compared the effects of two methods of training (eccentric training and concentric training) with untrained rats and showed that the mechanical properties of tendons in rats improve after specific training, especially following eccentric training. In a case-control study, Sánchez *et al*. [50] used autologous platelet-rich matrices (PRGF) during Achilles tendon surgery. They showed that athletes receiving PRGF recovered their range of motion earlier, showed no wound complication and took less time to take up gentle running and to resume training activities. In addition, they showed that the level of TGF-β1, PDGF-AB, VEGF, EGF and HGF significantly correlated with the number of platelets. Also, Filardo *et al*. [51] reported a partial tear of the Achilles tendon in a 34-year-old competitive athlete. They applied autologous platelet growth factors through multiple PRP injections; approximately 6.5 billion platelets were injected into the lesion three times, 7 days apart. The fast tissue repair, confirmed by magnetic resonance and ultrasound imaging, allowed a swift return to full functionality and competitive sports activity.

Recent investigations have shown that autogenous platelets may not be effective in tendon healing, as previously supposed. de Vos *et al*. [52] in a stratified, block-randomized, double-blind, placebo-controlled trial study showed that among patients with chronic Achilles tendinopathy who were treated with eccentric exercises, a PRP injection compared with a saline injection did not result in greater improvement in pain and activity. Also, de Vos *et al*. [53] in a double-blind, randomized, placebo-controlled clinical trial study showed that injecting PRP for the treatment of chronic midportion Achilles tendinopathy does not contribute to an increased tendon structure or alter the degree of neovascularisation, compared with placebo. Jo *et al*. [54] in a cohort study showed that application of PRP during arthroscopic rotator cuff repair did not clearly demonstrate accelerated recovery clinically or anatomically except for an improvement in internal rotation. Finally, Schepull *et al*. [28] in a randomized controlled trial injected autologous PRP with 10 times more platelet concentration than the physiologic plasma in patients with acute Achilles tendon ruptures and showed that PRP is not useful for treatment of Achilles tendon ruptures. Kaux and Crielaard [36] suggested that despite the proven efficacy of PRP on tissue regeneration in experimental studies, there is currently scanty tangible clinical evidence with respect to its efficacy in chronic tendon disorders.

The animal-based studies have used allogenous form of platelets for tendon healing. Aspenberg and Virchenko [34] in a rat Achilles tendon defect model showed that after intra-hematoma injection of allogenous concentrated platelets, the tendon callus strength and stiffness increased by ∼ 30% after 1 week, which persisted for as long as 3 weeks after the injection. Treatment improved callus maturation in the lesion. Virchenko *et al*. [27] in an Achilles tendon transection model in rat showed that the allogenous form of PG significantly increases the mechanical strength of the healing tendons after 14 days. They concluded that platelets and thrombin had independent and additive stimulatory effects on tendon repair. Beck *et al*. [55] also showed that allogenous PRP is effective in the healing and regeneration of tendon-from-bone supraspinatus tear model in rats because it increased fibroblastic response and vascular proliferation during the first 21 days of tendon injury. Sato *et al*. [26] also investigated the role of PG on the healing of intrasynovial flexor tendons in a rabbit model and showed that PG significantly increases biomechanical properties of the healing tendons after 2 and 3 weeks of tendon injury. Finally, Matsunaga *et al*. [56] using allogenous compact platelet-rich fibrin scaffold (CPFS), implanted the CPFS in the injured area of the partial patellar tendon defect model in rabbits and showed the ultimate failure load and stiffness were higher for the treated patellar tendon after 12 weeks than the controls.

Although the allogenous form of platelets was effective in tendon healing of animal-based studies, few controversies exist. Parafioriti *et al*. [37] injected allogenous form of PRP in Achilles tendon tear model in rat and showed that single injection of PRP appear not useful for Achilles tendon tear in rat. The concentration of their PRP was not clear. However, platelets were effective in most of the animal-based studies.

To date, few studies have used xenogenous-based platelets in tissue healing. In a series of relevant studies, it has been shown that xenogenous-based human platelets have promising curative effects on healing and regeneration of radial bone defect model in rabbit [29,35,39]. The results of the present study proved that the xenogenous form of platelets could be used in tendon tissue engineering approaches, but further studies are needed to answer whether the effectiveness of allogenous and xenogenous platelets in wound healing has correlation with platelet growth factors or it is due to the role of platelets on inflammation, or both.

PRPs used in the recent studies are much different from each other [28,53-57]. DeLong *et al*. [30] introduced a new PAW classification system for PRPs. The PAW classification system is based on three components: i) the absolute number of platelets; ii) the manner in which platelet activation occurs; and iii) the presence or absence of WBCs. It should be highlighted that the DeLong *et al*. classification was designed based on the autogenous form of PRP, while we used xenogenous-based bovine platelets. In addition, we removed the WBCs, while in their classification there is no designed class for those PRPs prepared without WBCs. The only thing we can classified our PG is the concentration of platelets and the activation method. Therefore, the term P4-X may be more suitable than P4-X-Bβ for our PG. It is possible to add a class negative (N) for the PAW classification system. In this class, N could be referred to: no WBCs. Two other classifications should also be designed: first, the source of the platelets (e.g., autogenous, allogenous and xenogenous) and second, the method of PRP production (e.g., commercial kit, laboratory devices). These two classes are important to be designed in order to compare the PRP and PGs used in all the human- and animal-based studies. Finally, the presence or absence of RBCs could be designed as a class. Our PRP did not have RBCs, too.

Combination of bovine platelets with three-dimensional CI is a novel approach for tendon regeneration. This choice is simple, inexpensive and effective approach, which could be considered as an alternative to current methods of tendon tissue engineering and regenerative medicine. As the availability and cost-effectiveness of the biomaterials are important in tissue engineering, this is the most important merit of bovine platelets which represent it as a suitable source for tissue engineering purposes, compared to the autogenous and allogenous sources. Although this study showed several important roles of bovine platelets on tendon healing, it should be highlighted that this is a preliminary study in rabbits and its results should not be extrapolated and generalized to the clinical setting. Before clinical application, it is strongly recommended to test subcutaneous biocompatibility of the PG-CI. Perhaps, bovine thrombin, platelets and collagen molecules should be tested for their adverse effects on human sensitivity; however, we did not come across any adverse reaction in rabbits. Although animal studies are important line of medical researches, animals have their own limitations which could be correlated with their anatomic, posture, habit, diet and other variations with the clinically ill patients [3].

## 5. Conclusion and future prospective

We successfully produced and applied bovine-pure PG embedded within the tissue-engineered CI in large Achilles tendon defect model in rabbits. *In vitro*, bovine platelets increased cell cytocompatibility and viability of the CI. *In vivo*, it increased the tenoinductivity, tenoconductivity, tenogenesis and tenoincorporation of the CI. In addition, the bovine platelets increased inflammation for a short period and thus enhanced fibroplastic response, which then resulted in a better remodeling during tendon healing. The treated tendons with bovine platelets embedded within the CI had superior structure and function than the controls. As availability and cost-effectiveness of biomaterials are important in tissue engineering, based on the results of the present study regarding the effectiveness of bovine PG on tendon healing, combination of bovine platelets with CI as one bioactive graft may be a valuable treatment strategy in the management of Achilles tendon defects in the clinical setting. However, the results of this novel but preliminary study should be confirmed in future studies.

## Declaration of interest

This work was supported by the Veterinary School, Shiraz University and the Iranian National Science Foundation (grant ISNF 87020247). The funders had no role in study design, data collection, analysis, decision to publish, or preparation of the manuscript. The authors have no other relevant affiliations or financial involvement with any organization or entity with a financial interest in or financial conflict with the subject matter or materials discussed in the manuscript apart from those disclosed.

## Supplementary Material

Supplementary 2: Scoring criteria
