# Supplementary material for: Effectiveness of xenogenous-based bovine-derived platelet gel embedded within a three-dimensional collagen implant on the healing and regeneration of the Achilles tendon defect in rabbits
Source: Expert Opin Biol Ther. 2014 May 19;14(8):1065–89. doi: 10.1517/14712598.2014.915305 (PMC4743604; doi:10.1517/14712598.2014.915305)
Supplement: Supplementary 2: Scoring criteria [file iebt_a_915305_sm0001.doc]

**Protocol S1**

1. **Preparation of the collagen implant**
   1. **Collagen electrospinning**

Collagen type I was extracted from the bovine superficial digital flexor tendon according to the methods of Foltran et al [1]. The purity of the type I collagen was assessed by SDS/PAGE using 6% separating gels. The gel was then stained overnight with 0.017% (w/v) Coomassie blue R-250 (Bio-Rad®, Hercules, USA) in 38.8% methanol and 6.8% acetic acid. Subsequently, the gel was destained with 5% methanol and 5% acetic acid for 48 hours.

The electrospinning solution was prepared by mixing collagen (type I acid soluble from bovine tendon) in acetic acid (>99%; EMD, San Diego, CA). The solution (7.5 % (w/w) collagen) was loaded into a syringe (Air-tite Products, Virginia Beach, VA) with a blunt-ended needle (18, gauge). The syringe was then placed on a syringe pump (New Era Pump Systems, Inc., NY) and directed toward a grounded collection device. Voltages of 6 kV were applied to the needle when the flow rate on the syringe pump was set to dispense at 0.15 mL/h. Uniformly aligned fibers were constructed by electrospinning onto a dual plate device. Dual plate devices used in this experiment consisted of two 2.5×0.5 cm copper strips attached to a gap substrate with Gluseal (Glustitch, Gulf Road Point Roberts, WA). The gap substrate was quartz glass (McMaster-Carr, Elmhurst, IL) with an electrical resistivity of 10^20^ Ωm. The gap between the copper strips was 1 cm, and each copper strip had a separate grounding wire. The dual plates were 4 cm away from the needle tip. The copper plates were removed after electrospinning [2]. Highly aligned and large electrospun collagen fibers with a diameter of 272 ± 183 nm were produced successfully (Fig. 1B, S1 and S2 A). The collagen matrix was then let to be dried at room temperature overnight to remove remaining solvents.

- 1. **Production of tridimensional collagen implant**

After electrospinning, acid-solubilized bovine tendon type I collagen was mixed with phosphate buffered saline (PBS) (Sigma, 10 ×) and NaOH (0.1N) to prepare a collagen solution with pH between 7.2 and 7.4 and concentration of 10 mg/ml. This mixture was immediately pipetted into a syringe and injected into a custom made chamber (10 × 10 × 10 cm) box containing electrospun collagen matrix. The box was placed in an incubator at 4^o^C for 48 hours to polymerize the collagen gel and to produce large fibers. The collagens were aligned under 12 Tesla magnetic fields (CRETA, Grenoble) during polymerization [3]. The range of the ﬁber diameters was 1.82 µm to 3.19 µm. The electrospun collagen matrix (2D) acted as a core with its fibro-conductive characteristics and improved alignment of the newly formed collagen fibers. The collagen composite was cut into several pieces of the same size and shape as the rabbit's Achilles apparatus (L=2cm, H=3. 5 mm, W=3 mm) (Fig. S1 and S2).

- 1. **Cross linking of the collagen implant**

For crosslinking, the implants were suspended in iso-osmolar 0.1% riboflavin solution with Dextrane T500 (as a photosensitizer), 20 minutes before the irradiation to allow sufficient saturation of the implant. The implants were irradiated for 60 minutes with UV (wavelength of 365 nm) at a working distance of 5 cm, with an irradiance of 3 mW/cm^2^ corresponding to a surface dose of 5.4 J/cm^2^, (UV-X^TM^, Peschke Meditrade, Cham, Switzerland). During irradiation, drops of riboflavin solution were applied to the implant every 4 to 5 minutes to sustain the necessary concentration of the riboflavin. The implants were neutralized with distilled water four times (30 min each) (Fig. S1) [4].

- 1. **Maintenance of the implants and sterility**

The final product was repeatedly washed with distilled water to remove the residual enzymes and chemical reagents, let to be dried overnight, received 100 Gray g-radiation for 120 min and then suspended in ethanol 96% to produce and maintain its sterility until surgery. Two hours before surgery, the implant was dried to evaporate the ethanol residues and repeatedly washed with distilled water (10 min×12 times) and finally suspended in isotonic saline solution to soften the implant for better surgical handling [5].

1. **Initial tests of the scaffold**
   1. **Morphology of the scaffolds**

Morphology of the scaffolds was studied by SEM (*n=10*). Porosity (free spaces between the collagen fibers) was deﬁned as the ratio of empty area to the total area [2] (Fig. 1).

- 1. **Microbiological tests**

The scaffolds were tested for sterility immediately after sterilization so that they were immersed in a Nutrient Agar Broth (NEOGEN Co. Lansing, MI, USA) to cultivate fastidious micro-organisms and maintained under agitation at 25 °C for 48 h. Non-sterile scaffolds (*n=10*) were used as negative control while the Graft jacket (Regenerative Tissue Matrix, Wright Medical Technology, Inc. San Antonio, TX, USA) was used as the positive control (*n=10*). Bioburden Challenge Test was also performed in a following manner: Test organisms that included Methicillin-resistant *Staphylococcus* *aureus* (MRSA; DMST 20645 lot no. 3273, NIH) and *Bacillus* *subtilis* (ATCC 6633 DMST 15896 lot no. 3479, NIH) were suspended in tryptic soy broth (TSB) (Sigma-Aldrich Co. LLC) to provide a final concentration of 10^2^ and 10^4^ cfu/mL. Inoculation of the test carrier was performed by using a micropipette to place 20 μL of the test suspension on the surface of scaffolds and left to dry in the incubator for 18 h at 37°C. The scaffolds were then sterilized as described before. After sterilization, the scaffolds were then transferred to test tubes containing TSB and were incubated at 25 °C for 7 days. The turbidity of the TSB was measured every day using a UV spectrophotometer at 625 nm wavelength and using McFarland Standards as a reference to estimate the number of colonies [6].

- 1. **Determination of endotoxin content**

The Limulus Amebocyte Lysate (LAL) (ToxinSensor^TM^ Gel Clot Endotoxin Assay Kit, GenScript Inc. Piscataway, NJ, USA) test was used according to the manufacturer's recommendation. The LAL reagent was mixed with the samples into the endotoxin-free vials, and then incubated at 37 °C for 60 minutes. Each vial was then inverted and checked whether a gel was formed.

- 1. **Cell seeding and cell culture**

Rat skin ﬁbroblasts (cell line CRL-1213) were obtained from the American Type Culture Collection (Manassas, VA) and cultured (37 ^o^C, CO_2_ 5%, pH=7.4) in Dulbecco’s Modiﬁed Eagle Medium supplemented with 10% fetal bovine serum, 20 U/ml penicillin, and 20 µg/ml streptomycin (Invitrogen, Carlsbad, CA). Cell culture medium was replaced every three days. Cells were passaged at conﬂuence and the 4–8^th^ passage ﬁbroblasts were used for the seeding. The sterile scaffolds were transferred into the wells of a custom-made 6-well culture chamber and seeded each with 1ml of 5.0 × 10^5^ cells.

The scaffolds were grown *in vitro* in a 5% CO_2_ incubator at 37 °C for 5, 10 and 20 days, with the medium being replaced every 3 days. The tissue constructs were removed from cell culture, and after routine histologic preparation were stained with hematoxylin and eosin and examined by a light microscope (Olympus, Tokyo, Japan) for regular cell counting and studying their distribution in the histopathologic fields. Number of cells per microscopic field in five fields per specimen (*n=10*) were counted [7,8].

- 1. **Cytocompatibility of the scaffolds**

Cell viability was determined by live/dead cell assay using ﬂuorescein diacetate (FDA, Molecular Probes, Invitrogen Corporation) (live) and propidium iodide (Cayman Chemical Company, Michigan, USA) (dead). The scaﬀolds with the ﬂuorescence stained cells were viewed under a Nikon ﬂuorescent microscope. A number of the live stained cells and dead stained cells were counted by the computer software (Image J, NIH, USA). The viability index was analyzed according to the following equation: Viability index= (number of viable cells/total number of cells) × 100 [8].

- 1. **Biomechanical testing of the scaffolds**

The un-cross-linked collagen implants, cross linked collagen implants and the hybridized collagen-PDS implants were subjected to the biomechanical test using the tensile testing machine (INSTRON® Tensile Testing Machine, London, UK). Scaffolds were hydrated for 24 h in PBS (pH 7.2) and drawn at a speed of 10 mm/min. The maximum load, maximum stress, strain and modulus of elasticity of the implants were extracted from the generated force-displacement and stress-strain curves [2].

- 1. **Determination of water binding capacity of the scaffolds**

Scaffold samples of about 5 mg dry weight were incubated in 3 ml PBS (pH 7.2) at 20^o^C. After 1 h, the wet weight was determined and the water-binding capacity calculated according to the following equation: water-binding capacity = (wet weight-dry weight) / (dry weight) [2,9].

1. ***In vitro* results**
   1. **Collagen purity**

Collagen extract contained two main α- chains (α_1_ and α_2_) and one β chain with very little contamination of other proteins. This suggested that collagen type I extract were pure with very little protein degradation product or other protein contamination (Fig. S3 A).

- 1. **Morphology of the scaffolds**

The transverse diameter of the electrospun fibers in 2D collagen matrix was 272 ± 183 nm (ranging 89 to 455 nm) (Fig. 1 B). Also the transverse diameter of the polymerized microfibers in the collagen gel (3D) was 2.54 ± 0.68 µm (ranging 1.82 to 3.19µm) (Fig. 1 C and D). In cross sections of the collagen implant, the porosity was 17.62 ± 4.88% (Fig. 1 E and F). The average diameter of these pores was 8.43 ± 4.27 µm (*n=100* pores). The proportion of the aligned collagen fibers/randomly formed collagen fibers were 71.27 ± 12.81/28.91 ± 4.22 (Fig. 1 C).

- 1. **Sterility of the scaffolds**

After 48 h incubation, the untreated controls (unsterilized scaffolds) produced signs of growth during sterility test whereas the Graftjacket and the sterilized scaffolds remained free of growth throughout the same time period and showed successful sterilization throughout the treatment durations. The result of bioburden test also showed that the sterilization technique was effective because the broth media was clear after challenging with high bacterial suspension even after 7 days incubation.

- 1. **Endotoxin level of the scaffolds**

The endotoxin levels of the collagen implants were below 0.25 EU/ml and no gel or turbidity was formed.

- 1. **Cell viability and cytocompatibility of the scaffolds**

Almost all of the fibroblasts were green, indicating the cells were live. Lack of propidium iodide (red) stained dead cells supports the idea that normal rat ﬁbroblasts were attached to the scaffold and that the majority of the cells were viable. A number of the proliferated cells in the scaffolds were 89.32 ± 12.84 (day 5), 202.17 ± 31.09 (day 10), and 358.92 ± 66.82 (day 20), at histology level (× 200) (Fig. S3B). The number of FDA stained viable cells in the scaffold (×200) was 84.71 ± 9.41 (day 5), 186.33 ± 27.91 (day 10), and 321.34 ± 49.23 (day 20) and was 94.83% (day 5), 92.16 % (day 10) and 89.52% (day 20) of the total cellularity (vitality index). The pattern of cell proliferation was homogenous in the scaffold (Fig. S3 C-E). The SEM images indicated that the fibroblasts well proliferated both on the surface and internal architecture of the implants and produced matrix (Fig. S3 F and G).

- 1. **Biomechanical characteristics of the scaffolds**

The maximum load ^N^ (95.37 ± 4.71^collagen-PDS^ vs. 28.33 ± 2.19 ^cross-linked collagen^ *vs*. 4.56 ± 1.92^un cross-linked collagen^, *P=0.001* for all), maximum stress ^N/mm2^ (9.08 ± 1.79 ^collagen-PDS^ vs. 2.69 ± 0.47 ^cross-linked collagen^ *vs*. 0.40 ± 0.11^un cross-linked collagen^, *P=0.001* for all), and modulus of elasticity ^kPa^ (610.46 ± 56.92 ^collagen-PDS^ *vs*. 43.81 ± 4.19 ^cross-linked collagen^ *vs*. 4.05 ± 1.28 ^uncross-linked collagen^) of the hybridized collagen-PDS implant (*n=10*) were significantly higher than those of the cross-linked (*n=10*) and un-cross-linked (*n=10*) collagen implants. Also the maximum strain% of the hybridized collagen-PDS implant (n=10) was significantly lower than those of the un-cross-linked (*n=10*) and cross-linked (*n=10*) collagen implants (14.81 ± 1.28 ^collagen-PDS^ *vs*. 61.34 ± 4.71 ^cross-linked collagen^ *vs*. 98.55 ± 7.23 ^uncross-linked collagen^, *P=0.001* for all).

- 1. **Water binding capacity of the scaffolds**

Water binding capacity of the un-cross-linked collagen scaffold (*n=10*), cross-linked collagen scaffold (*n=10*), collagen-PDS scaffold (*n=10*) and intact Achilles tendons of the rabbits (*n=10*) were 20.8 ± 1.8, 7.2±1.2, 1.8 ± 0.4 and 0.9 ± 0.2, respectively.

**References**

1. Foltran I, Foresti E, Parma B, Sabatino P, Roveri N. (2008) Novel biologically inspired collagen nanofibers reconstituted by electrospinning method. Macromol Symp 269: 111–118.
2. [Wray LS](http://www.ncbi.nlm.nih.gov/pubmed?term=Wray LS%5BAuthor%5D&cauthor=true&cauthor_uid=19193140), [Orwin EJ](http://www.ncbi.nlm.nih.gov/pubmed?term=Orwin EJ%5BAuthor%5D&cauthor=true&cauthor_uid=19193140). (2009) Recreating the microenvironment of the native cornea for tissue engineering applications. [Tissue Eng Part A](http://www.ncbi.nlm.nih.gov/pubmed?term=Recreating the Microenvironment of the Native Cornea for Tissue Engineering Applications)  15: 1463-1472.
3. [Dubey N](http://www.ncbi.nlm.nih.gov/pubmed?term=Dubey N%5BAuthor%5D&cauthor=true&cauthor_uid=11352087), [Letourneau PC](http://www.ncbi.nlm.nih.gov/pubmed?term=Letourneau PC%5BAuthor%5D&cauthor=true&cauthor_uid=11352087), [Tranquillo RT](http://www.ncbi.nlm.nih.gov/pubmed?term=Tranquillo RT%5BAuthor%5D&cauthor=true&cauthor_uid=11352087). (2001) Neuronal contact guidance in magnetically aligned fibrin gels: effect of variation in gel mechano-structural properties. [Biomaterials](http://www.ncbi.nlm.nih.gov/pubmed?term=Neuronal contact guidance in magnetically aligned) 22: 1065-1075.
4. [McCall](http://www.iovs.org/search?author1=A.+Scott+McCall&sortspec=date&submit=Submit) AS, [Kraft](http://www.iovs.org/search?author1=Stefan+Kraft&sortspec=date&submit=Submit) S, [Edelhauser](http://www.iovs.org/search?author1=Henry+F.+Edelhauser&sortspec=date&submit=Submit) HF, [Kidder GW](http://www.ncbi.nlm.nih.gov/pubmed?term=Kidder GW%5BAuthor%5D&cauthor=true&cauthor_uid=19643975), [Lundquist RR](http://www.ncbi.nlm.nih.gov/pubmed?term=Lundquist RR%5BAuthor%5D&cauthor=true&cauthor_uid=19643975), et al. (2010) Mechanisms of corneal tissue cross-linking in response to treatment with topical riboflavin and long-wavelength ultraviolet radiation (UVA). Invest Ophthalmol Vis Sci 51: 129-138.
5. [Stillaert FB](http://www.ncbi.nlm.nih.gov/pubmed?term=Stillaert FB%5BAuthor%5D&cauthor=true&cauthor_uid=18635258), [Di Bartolo C](http://www.ncbi.nlm.nih.gov/pubmed?term=Di Bartolo C%5BAuthor%5D&cauthor=true&cauthor_uid=18635258), [Hunt JA](http://www.ncbi.nlm.nih.gov/pubmed?term=Hunt JA%5BAuthor%5D&cauthor=true&cauthor_uid=18635258), [Rhodes NP](http://www.ncbi.nlm.nih.gov/pubmed?term=Rhodes NP%5BAuthor%5D&cauthor=true&cauthor_uid=18635258), [Tognana E](http://www.ncbi.nlm.nih.gov/pubmed?term=Tognana E%5BAuthor%5D&cauthor=true&cauthor_uid=18635258), et al. (2008) Human clinical experience with adipose precursor cells seeded on hyaluronic acid-based spongy scaffolds. Biomaterials 29: 3953e3959.
6. [Siritientong T](http://www.ncbi.nlm.nih.gov/pubmed?term=Siritientong T%5BAuthor%5D&cauthor=true&cauthor_uid=21671201), [Srichana T](http://www.ncbi.nlm.nih.gov/pubmed?term=Srichana T%5BAuthor%5D&cauthor=true&cauthor_uid=21671201), [Aramwit P](http://www.ncbi.nlm.nih.gov/pubmed?term=Aramwit P%5BAuthor%5D&cauthor=true&cauthor_uid=21671201). (2011) The effect of sterilization methods on the physical properties of silk sericin scaffolds. [AAPS Pharm Sci Tech](http://www.ncbi.nlm.nih.gov/pubmed?term=The Effect of Sterilization Methods on the Physical Properties of Silk Sericin Scaffolds) 12: 771-781.
7. Khanam N, Mikoryak C, Draper RK, Balkus KJ Jr. (2007) Electrospun linear polyethyleneimine scaffolds for cell growth. Acta Biomater 3: 1050–1059.
8. Zhao L, Burguera EF, Xu HH, Amin N, Ryou H, et al. (2010) Fatigue and human umbilical cord stem cell seeding characteristics of calcium phosphate-chitosan-biodegradable fiber scaffolds. Biomaterials 31: 840–847.
9. Tsai SP, Hsieh CY, Hsieh CY, Wang D, Huang LL, et al. (2007) Preparation and Cell Compatibility Evaluation of Chitosan/Collagen Composite Scaffolds Using Amino Acids as Crosslinking Bridges. J Appl Polym Sci 105: 1774-1785.


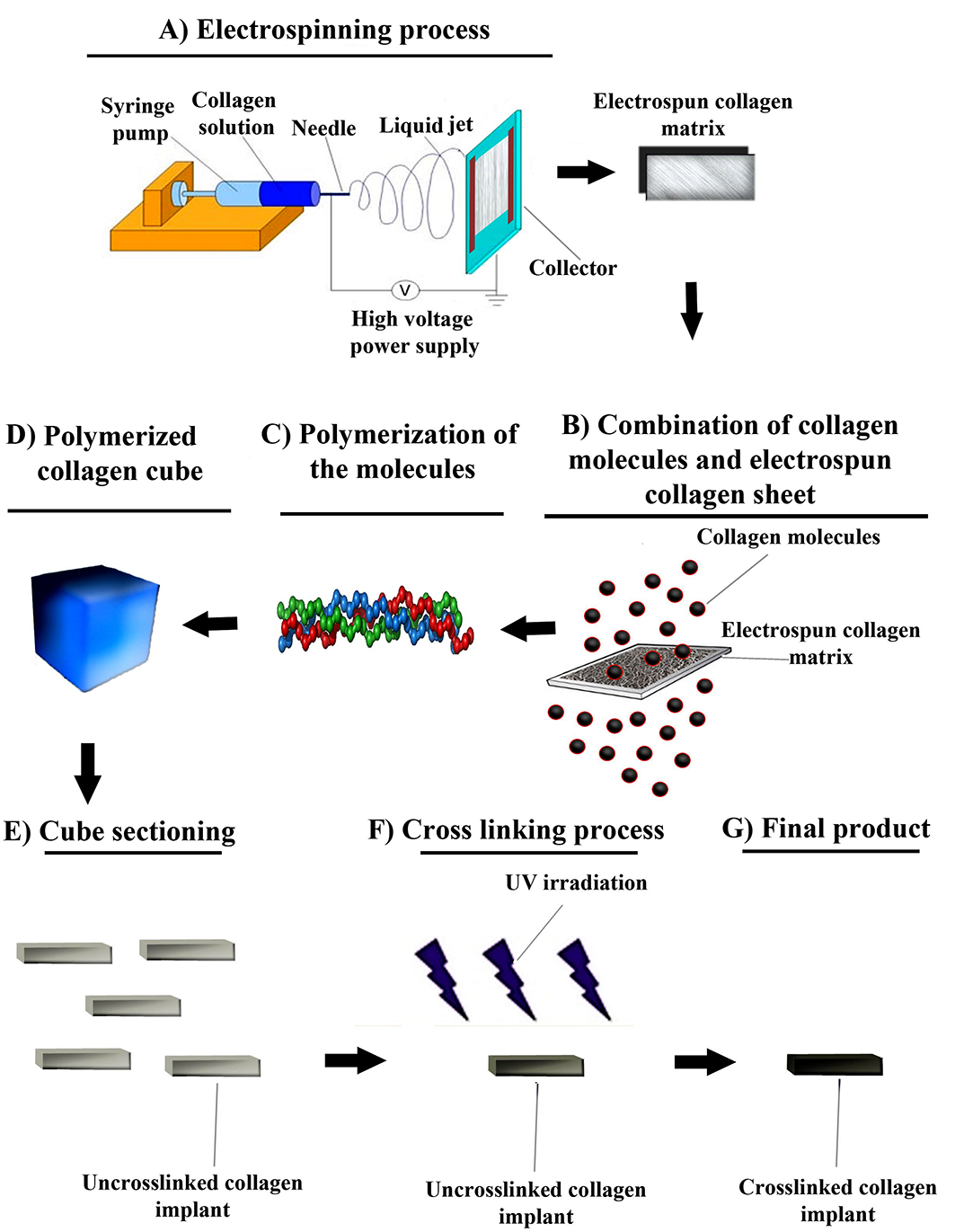


**Figure S1 Preparation of the collagen implant (Part 1).** The collagen solution was placed in the syringe pump with the needle charged at 6 kV with respect to the base plate (A). The nanofibers were harvested and mixed with fresh collagen solution (B), incubated for final polymerization (C). This hybridized collagen gel was dried (D) and cut into rectangular strips to form several prostheses (E) which were cross-linked (F), sterilized and then dried (G). Each arrow shows a next step (A to G).


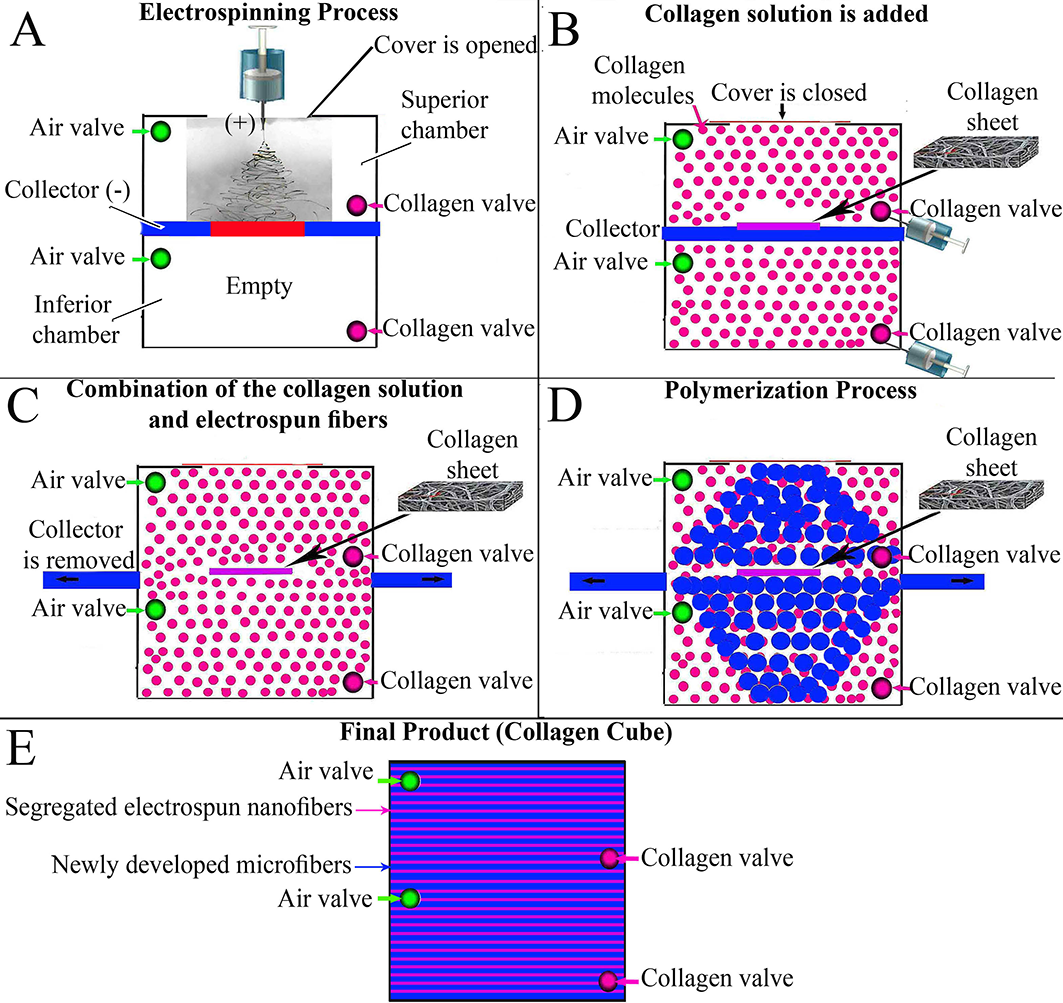


**Figure S2** **Preparation of the collagen implant (Part 2).** (A) The electrospun collagen nanofibers are collected in the gap collector (Arrows). B: after the electrospinning, the door of the box closed and the collagen solution was added through the collagen valve both over and under the collector. (C) The collector has been removed and the collagen solution and the electrospun collagen fibers were combined together. (D) The box was placed at the 4^oC^ for 48 hours to produce large fibers. The polymerization of the larger collagen fibers is started close to the electrospun collagen sheet. (E) The polymerization is completed and the nano and micro collagen fibers are formed so that the hybridized collagen cube is seen. The electrospun collagen matrix acted as a scaffold for the newly polymerized collagen fibers and improved their alignment along its fiber orientation. An electromagnetic field was applied during the polymerization in order to improve the final alignment of the fibers.


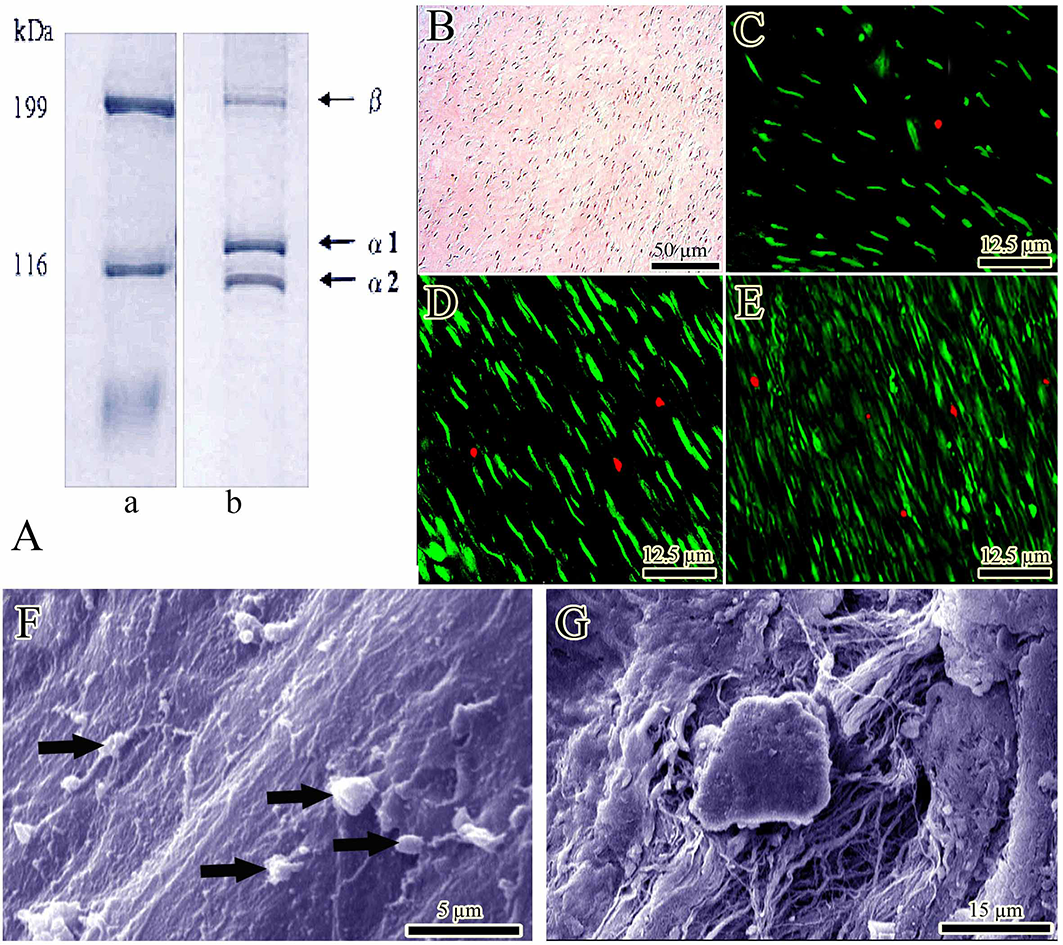


**Figure S3 *in vitro* findings.** (A) SDS-PAGE images of type I collagen using 6% poly-acrylamide gel to examine the purification of collagen extracts. Lane a: molecular marker, lane b: bovine collagen. (B) Histologic section of the constructs after 20 days of cell culture. The proliferating fibroblasts are infiltrated to the implant and proliferated. (C-E) Cell viability was determined by live/dead cell assay using ﬂuorescein diacetate (live) and propidium iodide (dead). (C, D and E) shows day 5, 10 and 20 after cell seeding. Almost all of the fibroblasts are green, indicating the cells are live. The lack of Propidium iodide stained dead cells (red) supports the idea that normal rat ﬁbroblasts have attached to the scaffold and that the majority of the cells are viable. (F and G) Surface and inside of the collagen implant after 20 days of cell seeding, respectively. The cells proliferated (arrows) and produced matrix.

**Table S1: Clinical scoring criteria**

| **The tarsal flexion degree of the injured leg compared to the normal leg, both in the cage and on the floor** | | | | | | | |
| --- | --- | --- | --- | --- | --- | --- | --- |
| **Score** | **Between legs** | | | | **Estimation of the degree** | | **Condition** |
| **0** | - Equal | | | | - 75-90 | | Normal |
| **1** | - Non equal | | | | - 50-74 | | Mild |
| **2** | - Non equal | | | | - 30-50 | | Moderate |
| **3** | - Non equal | | | | - >30 | | Severe |
| **4** | - Non equal | | | | - <15 | | Extensively severe |
| **Weight distribution of each animal on the hind limbs, in the cage and on the floor** | | | | | | | |
|  | **Weight distribution between limbs** | **Weight distribution between hind legs** | | **The most weight bearing legs** | | **The injured left hind leg condition** |  |
| **0** | - Mostly hind limb | - Equal | | - Both hind limbs | | - Weight bearing | Normal |
| **1** | - Mostly hind limb | - Not equal | | - Right hind limb | | - Weight bearing | Mild |
| **2** | - Mostly forelimbs | - Not equal | | - Both forelimbs & right normal hind limb | | - Weight bearing | Moderate |
| **3** | - Not Equal | - Not equal | | - Both forelimbs and right normal hind limb | | - Non weight bearing | Severe |
| **4** | - Not Equal | - Not equal | | - Non weight bearing (sternal recumbency) | | - Non weight bearing | Extremely severe |
| **Pain in palpation of the injured area and pain in complete extension of the injured leg** | | | | | | | |
| **0** | - No reaction | | | | | | Normal |
| **1** | - Occasional vocalization | | | | | | Mild |
| **2** | - Frequent vocalization | | | | | | Moderate |
| **3** | - Vociferous vocalization, withdraw limb, bites, struggles | | | | | | Severe |
| **Heel and toe position of the injured leg (left hind paw)** | | | | | | | |
|  | **Heel** | | **toe** | | | |  |
| **0** | - Up | | - Down | | | | Normal |
| **1** | - Near the floor (up) | | - Down | | | | Mild |
| **2** | - Down | | - Down | | | | Moderate |
| **3** | - Down | | - Up | | | | Severe |
| **Swelling at the injured area (left hind paw)** | | | | | | | |
| **0** | Is not tender, warm and bowed | | | | | | Normal |
| **1** | Slightly warm and bowed, color is not changed | | | | | | Mild |
| **2** | Tenderness, bowed and completely warm. Color in not changed | | | | | | Moderate |
| **3** | Obvious tenderness, bowed and warm. Color changed. | | | | | | Severe |

**Table S2: Ultrasonographical scoring criteria**

| **Score** | 1. **Echogenicity** | 1. **Hyper echogenic area / hypo echogenic area of the tendons (homogeneity)** | 1. **Transverse movement of the tendon (Index for peritendinous adhesion)** |
| --- | --- | --- | --- |
| **0** | - Normal echogenicity | - Smooth (homogenous) echogenicity | - Free to move |
| **1** | - Slightly hyper-echoic | - Non-smooth (heterogeneous) echogenicity (mild) | - Movable in one direction (left or right) |
| **2** | - Hyper-echoic | - Non-smooth echogenicity (moderate) | - Movable in one direction with force (left or right) |
| **3** | - Hypo-echoic | - Amputated view or non-smooth echogenicity (severe) | - Fixed or non-movable |
|  | 1. **Diameter of the injured tendon / intact contralateral (regeneration volume)** | 1. **Diameter of the peritendinous low echogenic area / diameter of the high echogenic tendon (intensity of the peritendinous adhesion)** | 1. **Diameter of the proximal part of the tendon / diameter of the distal part of the tendon (regenerative proportion)** |
| **0** | - 100% | - Less than 10% | - 110-140% |
| **1** | - 90% | - 11-25% | - 91-109% |
| **2** | - 101-110% | - 26-50% | - 75-90% |
| **3** | - 75%-100% | - 50-100% | - 50-74% |
| **4** | - 50%-74% | - 100-150% | - 25-49% |
| **5** | - 0-50% | - 151% and more | - Less than 25% |

Table S3: Gross morphological scoring criteria

| **Gross pathological findings (visual observation)** | | | |
| --- | --- | --- | --- |
| **Score** | 1. **Peritendinous adhesion** | 1. **Hyperemia** | **Status** |
| **0** | - No adhesion | - No hyperemia, shiny glistening surface appearance | Normal |
| **1** | - Tendon was easily detached from the surrounding tissues by blunt dissection | - Only in the paratenon | Mild |
| **2** | - For detachment from the surrounding tissues, tendon needed little sharp dissection | - It was extended to the tendon proper but it was not severe in nature | Moderate |
| **3** | - For detachment from the surrounding tissues, tendon needed completely sharp dissection | - It was extensively extended to the tendon proper and made its appearance more pink and dark | Severe |
| **Score** | 1. **General appearance** | **4) Muscle Atrophy** | **Status** |
| **0** | - Tendon is a unit structure (the tendinous tissue is organized as a separate tissue and could be differentiated from the surrounding structure) and is continued between the gastrocnemius muscle and calcaneal tuberosity with the same diameter and homogeneity | - The transvers diameter of the largest bulk of the muscle is more than or equal to 350% of the transvers diameter of the largest part of the Achilles tendon at its mid part. | Normal |
| **1** | - Same as above but the diameter of the injured area is larger than the proximal and distal parts of the tendon. The tendon generally is a unit structure. | - The transvers diameter of the largest bulk of the muscle is more than or equal to 250% of the transvers diameter of the largest part of the Achilles tendon at its mid part. | Mild |
| **2** | - Same as above but the diameter of the injured area is lower than the proximal and distal parts of the tendon. The tendon generally is a unit structure. | - The transvers diameter of the largest bulk of the muscle is more than or equal to 200% of the transvers diameter of the largest part of the Achilles tendon at its mid part. | Fairly moderate |
| **3** | - The injured area of the tendon is not a unit structure but the proximal and the distal parts of the tendon are a unit structure. | - The transvers diameter of the largest bulk of the muscle is more than or equal to 150% of the transvers diameter of the largest part of the Achilles tendon at its mid part. | Moderate |
| **4** | - The whole of the tendon is not a unit structure. No structure similar to tendon is seen between the gastrocnemius muscle and calcaneal tuberosity and the posterior tibialis tendon is seen. In normal condition Achilles tendon covers the posterior tibialis muscle but in this condition due to the lysis of the Achilles tendon the posterior tibialis muscle could be seen. | - The transvers diameter of the largest bulk of the muscle is more than or equal to 10% of the transvers diameter of the largest part of the Achilles tendon at its mid part. | Severe |
| **Score** | **5) Muscle fibrosis** | | **Status** |
| **0** | - No fibrosis is seen in the gastrocnemius muscle and the tendinous portion of the Achilles is the only connective tissue that covered the muscle | | Normal |
| **1** | - Mild fibrosis is seen in the muscle but more than 75% of the muscle has red color and had gross appearance similar to the muscle | | Mild |
| **2** | - Between 50 to 74% of the muscle has the characteristics of the muscle but the fibrosis is significant | | Moderate |
| **3** | - More than 50% of the muscle shows fibrosis and the fibrous tissue filled the spaces between muscle fibers. | | Severe |
| **4** | - No muscular characteristic could be seen in the gastrocnemius muscle because all of the muscle was substituted by fibrous tissue | | Extremely severe |

Table S4: Histological scoring criteria

| **Histopathologic analysis** | | | |
| --- | --- | --- | --- |
| **Score** | **1) Alignment** | **2) Perivascular edema** | **Status** |
| **0** | - Collagen fibers were longitudinally oriented in only one direction and the tenoblasts and tenocytes were laid longitudinally along their orientation | - No edema | Normal |
| **1** | - Collagen fibers were longitudinally oriented in one direction pattern but there were few areas of unorganized collagen fibers in the field | - Presence of edema just around small vessels | Mild |
| **2** | - Collagen fibers were not longitudinally oriented and the irregular orientation pattern was predominant | - Presence of edema around small and medium sized vessels | Moderate |
| **3** | - There was no obvious pattern and the collagen fibers were disorganized | - Presence of edema around all types of vessels | Severe |
| **Score** | **3) Tissue Maturity** | | **Status** |
|  | **A) the appearance of the collagen fibers** | **B) cellular populations** |  |
| **0** | - More than 75% collagen fibers are dense and they have large size | - More than 75% are fibrocytes | Normal or near normal |
| **1** | - More than 50% of the collagen fibers are dense and they are of large size | - More than 50% are fibrocytes | Highly mature |
| **2** | - More than 25% of the collagen fibers are dense and they are medium sized | - More than 25% are fibrocytes | Moderately mature |
| **3** | - The collagen fibers are not dense but they are medium sized | - More than 75% are fibroblasts | Immature |
| **4** | - The collagen fibers are not dense and they are of small sized | - The inflammatory cells are predominant | Highly immature |
| **Score** | **4) Crimp pattern** | **5) Vascularity (at remodeling stage)** | **Status** |
| **0** | - More than 75% of the collagen fibers in the light microscopic field are wavy | - No vascular structures are visible in the tissue sections. | Normal |
| **1** | - 50%-75% of the collagen fibers in the light microscopic field are wavy | - Less than 10% of the tissue density belongs to vessels. | Optimum (remodeled) |
| **2** | - 25%-50% of the collagen fibers in the light microscopic field are wavy | - Less than 25% of the tissue density belongs to vessels | Early remodeling  (fairly acceptable) |
| **3** | - Less than 25% of the collagen fibers in the light microscopic field are wavy | - Less than 50% of the tissue density belongs to vessels | Fibroplasia (bad) |
| **4** | - No crimp pattern is seen | - Less than 75% of the tissue density belongs to vessels | Early fibroplasia or degenerative changes (extremely bad) |
| **5** |  | - More than 75% of the tissue density belongs to vessels | Healing is not in progress and the newly regenerated tissue is only vascularized. |

**Table S5: Base scoring system used for defining the ultrastructure analysis (SEM)**

| **1) Alignment** | | | | | |
| --- | --- | --- | --- | --- | --- |
| **Score** | **Status** | | **Directions of the collagen fibrils** | | **Direction of the fibroblasts and fibrocytes** |
| **0** | Near normal | | - Most of them are aligned in one directions | | - Most of them are laid in a direction of collagen fibrils |
| **1** | Highly aligned | | - More than ¾ of the collagen fibrils are aligned in one directions | | - More than ¾ of the cells are laid in a direction of collagen fibrils |
| **2** | Moderately aligned | | - More than ½ of the collagen fibrils are aligned in one directions | | - More than ½ of the cells are laid in a direction of collagen fibrils |
| **3** | Fairly aligned | | - More than ¼ of the collagen fibrils are aligned in one directions | | - More than ¼ of the cells are laid in a direction of collagen fibrils |
| **4** | Amorphous | | - Collagen fibrils are not aligned in one direction. | | - Most of the cells are not laid in the direction of the collagen fibrils |
|  | **2) Maturity of the collagen fibrils** | | | | |
| **Score** | **Status** | **Description** | | | |
| **0** | Normal | - Collagen fibrils are distributed in multimodal pattern. Minimum of five different category of fibril’s diameter (extremely small (0-64nm), Small (65-102nm), medium (103-153 nm), large (154-256nm), extremely large (257-307nm)) is seen at ultra-micrographs. | | | |
| **1** | Highly matured | - Collagen fibrils are distributed in multimodal pattern. Four different category of fibril’s diameter (extremely small (0-64nm), Small (65-102nm), medium (103-153nm), large (154-256nm)) is seen at ultra-micrographs. | | | |
| **2** | Matured | - Collagen fibrils are distributed in multimodal pattern. Three different category of fibril’s diameter (extremely small (0-64nm), Small (65-102), medium (103-153nm)) is seen at ultra-micrographs. | | | |
| **3** | Immature | - Collagen fibrils are distributed in bimodal pattern. Two different category of fibril’s diameter (extremely small (0-64nm), Small (65-102nm)) is seen at ultra-micrographs. | | | |
| **4** | Highly immature | - Collagen fibrils are distributed in unimodal pattern. Only one category of fibril’s diameter (extremely small (0-64nm) is seen at ultra-micrographs. | | | |
| **Score** | **3) Crimp pattern** | | |  |  |
| **0** | - More than 75% of the collagen fibers in the field are wavy | | |  |  |
| **1** | - 50%-75% of the collagen fibers in the field are wavy | | |  |  |
| **2** | - 25%-50% of the collagen fibers in the field are wavy | | |  |  |
| **3** | - Less than 25% of the collagen fibers in the field are wavy | | |  |  |
| **4** | - No crimp pattern is seen | | |  |  |
